# Assessment of an Antibody-in-Lymphocyte Supernatant Assay for the Etiological Diagnosis of Pneumococcal Pneumonia in Children

**DOI:** 10.3389/fcimb.2019.00459

**Published:** 2020-01-17

**Authors:** Michael J. Carter, Pallavi Gurung, Claire Jones, Shristy Rajkarnikar, Rama Kandasamy, Meeru Gurung, Stephen Thorson, Madhav C. Gautam, Krishna G. Prajapati, Bibek Khadka, Anju Maharjan, Julian C. Knight, David R. Murdoch, Thomas C. Darton, Merryn Voysey, Brian Wahl, Katherine L. O'Brien, Sarah Kelly, Imran Ansari, Ganesh Shah, Nina Ekström, Merit Melin, Andrew J. Pollard, Dominic F. Kelly, Shrijana Shrestha

**Affiliations:** ^1^Oxford Vaccine Group, Department of Paediatrics, University of Oxford, Oxford, United Kingdom; ^2^NIHR Oxford Biomedical Research Centre, Oxford, United Kingdom; ^3^Patan Academy of Health Sciences, Kathmandu, Nepal; ^4^School of Life Course Sciences, King's College London, London, United Kingdom; ^5^Wellcome Centre for Human Genetics, University of Oxford, Oxford, United Kingdom; ^6^Department of Pathology, University of Otago, Christchurch, Christchurch, New Zealand; ^7^Department of Infection, Immunity and Cardiovascular Disease, University of Sheffield Medical School, Sheffield, United Kingdom; ^8^International Vaccine Access Center, Department of International Health, Johns Hopkins Bloomberg School of Public Health, Baltimore, MD, United States; ^9^Expert Microbiology Unit, National Institute for Health and Welfare (THL), Helsinki, Finland

**Keywords:** pneumococcus (*Streptococcus pneumoniae*), pneumonia, antibodies, diagnostic test (MeSH), lymphocytes

## Abstract

New diagnostic tests for the etiology of childhood pneumonia are needed. We evaluated the antibody-in-lymphocyte supernatant (ALS) assay to detect immunoglobulin (Ig) G secretion from *ex vivo* peripheral blood mononuclear cell (PBMC) culture, as a potential diagnostic test for pneumococcal pneumonia. We enrolled 348 children with pneumonia admitted to Patan Hospital, Kathmandu, Nepal between December 2015 and September 2016. PBMCs sampled from participants were incubated for 48 h before harvesting of cell culture supernatant (ALS). We used a fluorescence-based multiplexed immunoassay to measure the concentration of IgG in ALS against five conserved pneumococcal protein antigens. Of children with pneumonia, 68 had a confirmed etiological diagnosis: 12 children had pneumococcal pneumonia (defined as blood or pleural fluid culture-confirmed; or plasma CRP concentration ≥60 mg/l and nasopharyngeal carriage of serotype 1 pneumococci), and 56 children had non-pneumococcal pneumonia. Children with non-pneumococcal pneumonia had either a bacterial pathogen isolated from blood (six children); or C-reactive protein <60 mg/l, absence of radiographic consolidation and detection of a pathogenic virus by multiplex PCR (respiratory syncytial virus, influenza viruses, or parainfluenza viruses; 23 children). Concentrations of ALS IgG to all five pneumococcal proteins were significantly higher in children with pneumococcal pneumonia than in children with non-pneumococcal pneumonia. The concentration of IgG in ALS to the best-performing antigen discriminated between children with pneumococcal and non-pneumococcal pneumonia with a sensitivity of 1.0 (95% CI 0.73–1.0), specificity of 0.66 (95% CI 0.52–0.78) and area under the receiver-operating characteristic curve (AUROCC) 0.85 (95% CI 0.75–0.94). Children with pneumococcal pneumonia were older than children with non-pneumococcal pneumonia (median 5.6 and 2.0 years, respectively, *p* < 0.001). When the analysis was limited to children ≥2 years of age, assay of IgG ALS to pneumococcal proteins was unable to discriminate between children with pneumococcal pneumonia and non-pneumococcal pneumonia (AUROCC 0.67, 95% CI 0.47–0.88). This method detected spontaneous secretion of IgG to pneumococcal protein antigens from cultured PBMCs. However, when stratified by age group, assay of IgG in ALS to pneumococcal proteins showed limited utility as a test to discriminate between pneumococcal and non-pneumococcal pneumonia in children.

## Introduction

Pneumonia is the leading cause of childhood mortality after the neonatal period, yet the pathogen-specific etiology of childhood pneumonia remains poorly defined (Liu et al., 2016; Feikin et al., 2017b). Using data from randomized controlled vaccine trials, vaccine-probe studies help reveal the pathogen-specific burden of disease by estimating the difference in disease between vaccinated and unvaccinated individuals (Feikin et al., 2014). Results from these studies estimate that approximately one third of children with pneumonia and radiographic consolidation have pneumococcal pneumonia in settings prior to the introduction of vaccines that prevent etiology-specific pneumonia, regardless of geography (O'Brien et al., 2009; Wahl et al., 2018). However, microbiological data to support this prevalence estimate are lacking. Accurate diagnostic tests for the etiology of pneumonia are needed to assess the etiology of pneumonia in changing epidemiological contexts, notably following vaccine implementation (Feikin et al., 2017b).

Direct aspiration of infected (lung) tissue is rarely used due to perceived safety concerns (Ideh et al., 2011; Howie et al., 2014), while culture of broncho-alveolar lavage samples is only possible on samples from children requiring mechanical ventilation. Culture of bacteria from blood is therefore the most widely used test for bacterial pneumonia in children admitted to hospital. However, estimates of yield from “true positive” bacterial pneumonia cases are <20% (Cutts et al., 2005), depending on prior antibiotic exposure, sample volume and culture technique (Driscoll et al., 2017). In the recent Pneumonia Etiology Research for Child Health (PERCH) case-control study, quantitative (q)PCR of *lytA* to determine whole blood pneumococcal load (Deloria Knoll et al., 2017), and density of nasopharyngeal (NP) colonization with *S. pneumonia* (Baggett et al., 2017), demonstrated only moderate ability to discriminate between pneumococcal pneumonia and age-matched community children.

An alternative approach to the diagnosis of pneumococcal pneumonia is to assess the immune response to the pathogen. Unfortunately, serological assays have limited specificity in the acute phase, or require convalescent samples to discriminate from past infections (Tuerlinckx et al., 2013; Andrade et al., 2016). We hypothesized that we could combine the etiological specificity of serological assays to a time-specific population of B cells (plasmablasts), that circulate during active infection (Carter et al., 2017), using the antibody-in-lymphocyte supernatant (ALS) assay.

The ALS assay was originally developed to assess vaccine-induced serological responses, and has since been developed for the diagnosis of enteric fever and tuberculosis (Chang and Sack, 2001; Sheikh et al., 2009; Darton et al., 2017b; Sariko et al., 2017). This assay is based upon testing the secretions of lymphocytes that are incubated *in vitro* following sampling from an unwell patient (without *ex vivo* stimulation). Following incubation, harvested supernatant can be tested for pathogen-specific antibodies using standard serological techniques.

We assessed the diagnostic performance of the ALS assay for the diagnosis of pneumococcal infection in a prospective study of childhood pneumonia in Nepal, a low income country in South Asia with a high burden of childhood pneumonia (Ministry of Health Population (MOHP) et al., 2012). We used five pneumococcal proteins as target antigens (choline binding protein A, CbpA; protein for cell wall separation of group B streptococci, PcsB; pneumococcal histidine triad D, PhtD; pneumolysin, Ply; serine threonine kinase protein C, StkpC). These antigens are thought to be expressed by all pathogenic pneumococci, are specific to pneumococci or closely related species, and have been used to assess the serological response to pneumococcal pneumonia (Andrade et al., 2014, 2016; Borges et al., 2016).

## Materials and Methods

### Ethics Statement

This prospective study was carried out in accordance with the protocol and the International Conference on Harmonization Good Clinical Practice standard. Literate parents/legal guardians all gave informed written consent prior to enrolment. Non-literate parents/legal guardians gave verbal and thumbprint consent in the presence of a literate (non-hospital/research staff) witness who could attest to the explanation of the patient information leaflet and the agreement of the signatory. The study protocol was approved by the Nepal Health Research Council (286/2014) and the Oxford Tropical Research Ethics Committee (09/15).

### Study Site and Population

Patan Hospital is in the Lalitpur sub-Metropolitan district of Nepal, contiguous with the city of Kathmandu. It is one of the largest hospitals in the country, and one of few with inpatient pediatric and pediatric critical care facilities. During the study period, the primary infant vaccination schedule included diphtheria-tetanus-pertussis, bacille-Calmette-Guérin, hepatitis B, *Haemophilus influenzae* type b (from 2009), oral and inactivated poliovirus and measles, rubella and Japanese encephalitis antigens, with 83% coverage for all antigens in 2011 in the Central Development Region of Nepal (including Lalitpur) [Ministry of Health Population (MOHP) et al., 2012]. Ten-valent pneumococcal conjugate vaccination (10-valent PCV) was introduced to the infant immunization schedule in Lalitpur in August 2015 at 6 and 10 weeks, and 9 months of age. There was a limited PCV catch-up campaign among infants.

### Enrolment and Sampling

**Figure 1:**
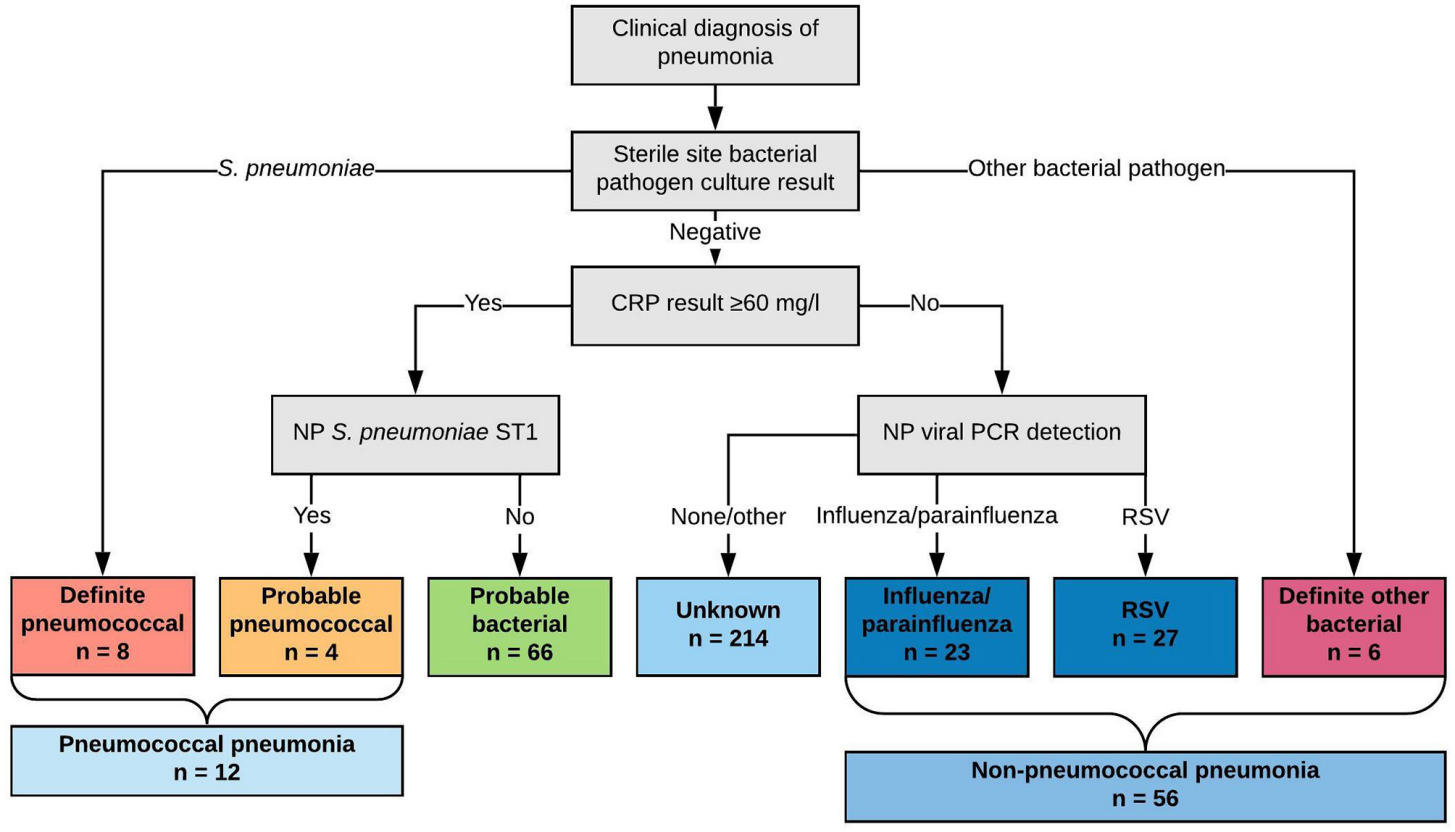
Classification of children by likely etiology of pneumonia into comparator groups (*definite pneumococcal, probable pneumococcal, probable bacterial, unknown, influenza/parainfluenza virus, RSV*, and *definite other bacterial*) and by diagnosis of “*pneumococcal pneumonia*” and “*non-pneumococcal pneumonia*”.

Children were enrolled for this study between 22nd December 2015 and 30th September 2016. Children were eligible for enrolment if ≥60 days of age and <15 years of age and being admitted to Patan Hospital with a clinical diagnosis of pneumonia. A small number of patients were diagnosed with pneumonia, enrolled, and then discharged directly before admission; their data are included here (Figure 1). The diagnosis of pneumonia was made by admitting pediatricians, reviewed by a consultant (attending) pediatrician, and was typically prior to results from radiographs or blood tests becoming available. Children who did not have a clinical diagnosis of pneumonia were excluded from the study. A digitalized radiograph was obtained on all children on study enrolment. All radiographs were independently interpreted using standardized WHO criteria (Liu et al., 2016) as endpoint consolidation, other infiltrate, or no consolidation/effusion/infiltrate by two specific readers (a pediatrician, and a radiologist). A second specific radiologist arbitrated upon all discordant results, and 10% of other radiographs. Radiographic findings were not used to exclude children from the study. Child healthy controls were enrolled from a vaccine clinic at 10 months of age, and all controls had been vaccinated with three doses of 10-valent PCV (most recent dose received ≥28 days prior to sampling).

Enrolled cases and controls all had 2 ml of blood sampled into heparinized and sterile centrifuge tubes for this study by peripheral venepuncture within 48 h of admission. In addition all enrolled children had full blood count, plasma (for storage) and inoculation of blood into Bactec Peds Plus culture bottles (Becton Dickinson, BD; USA) for automated incubation (Bactec, BD), subculture and identification of isolated organisms. Heparinized blood was immediately taken to the Microbiology Laboratory and processed within 4 h of sampling as described below. C-reactive protein (CRP) concentrations were measured following shipment of plasma to Oxford at Oxford University Hospitals NHS Foundation Trust.

A single flocked swab (Thermo Fisher Scientific, UK) was used to sample the nasopharynx, and a digital chest radiograph was taken, at admission. The NP swab was immediately and aseptically placed into skim-milk-tryptone-glucose-glycerin media and transported to the on-site Microbiology Laboratory, cultured for pneumococci, and subjected to Quellung serotyping. NP swabs in STGG media were subsequently stored at −80°C before transport to the UK on dry ice and further storage at −80°C. All chest radiographs underwent blinded review for radiographic endpoint consolidation by two clinicians (a pediatrician and a radiologist) according to World Health Organization criteria (Cherian et al., 2005), with quality control and discordant readings arbitrated upon by a second radiologist.

### PCR Methods

NP swabs were defrosted and DNA was extracted from 200 μl STGG media using the QIAGEN DNeasy 96 kit (QIAGEN, UK) using a modified protocol. Extracted DNA was subsequently transported to Micropathology Ltd (Warwick, UK) where samples (40 μl extracted DNA) were analyzed using the NxTAG Luminex Respiratory Pathogen Panel (Luminex Corp, USA) (Tang et al., 2016) according to manufacturer's instructions for: influenza A, influenza A H1, influenza A H3, influenza B, RSV A, RSV B, parainfluenza 1–4, coronaviruses 229E/NL63/OC43/HKU1, human metapneumovirus, rhinovirus/enterovirus, adenovirus, human bocavirus, *Chlamydophila pneumoniae, Legionella pneumophila*, and *Mycoplasma pneumoniae*. Results were reported as positive or negative for each pathogen. We considered RSV (any group), influenza virus (any serotype) or any of the parainfluenza viruses 1–4 as pathogenic, since these were highly associated with case status in the PERCH study, and were prevalent (≥5 cases for each virus) in our cohort.

### Antibody-in-Lymphocyte Supernatant Assay

Samples of fresh, heparinized, whole blood were separated by centrifugation over Ficoll media with a density of 1.077 g/ml (Histopaque 1077, Sigma-Aldrich, USA) at 400 g for 20 min with minimal acceleration and deceleration. This yielded plasma, peripheral blood mononuclear cells (PBMCs) and a sediment of red cells and polymorphonuclear cells. The PBMC layer was manually aspirated and washed by resuspension and centrifugation twice into RPMI culture media plus penicillin (500 U/l), streptomycin (0.25 mg/l), and L-glutamine (10 mmol/L; all Sigma-Aldrich; “R0 medium”). Following manual counting and calculation of the total number of PBMCs, the cells were resuspended into R0 medium plus 10% fetal bovine serum (heat-inactivated and sterile-filtered, Sigma-Aldrich; “R10 medium”), and incubated in a sterile cell culture plate (Greiner, Germany) at 37°C and 5% CO_2_ for 48 h. Following incubation the cell suspension was separated by centrifugation with the resulting supernatant (ALS) preserved with 40X protease inhibitors (25 μl per ml of ALS) and stored immediately at −80°C before shipping to Oxford and Helsinki on dry ice for further analyses.

A fluorescent multiplexed bead-based immunoassay (FMIA) to detect IgG ALS to five pneumococcal proteins (CbpA, PcsB, PhtD, Ply, and StkpC) was used for this study as previously described (Andrade et al., 2014, 2016). In brief, samples of ALS were diluted to 1/25 in phosphate buffered saline containing 10% fetal bovine serum, with the FMIA performed as a 5-plex assay with 1,200 beads per region per well. IgG in ALS were detected using RPE-conjugated goat anti-human IgG (Jackson Immunoresearch, USA). Pneumococcal reference standard serum 007sp (NIBSC, UK) was used as the reference with an arbitrary assigned concentration of 100 units/ml for each anti-pneumococcal antibody.

### Classification of Childhood Pneumonia Etiology by Comparator Group

We classified children by comparator groups based on likely etiology of pneumonia (where these comparator groups are used in the text, they are italicized for clarity.) This classification scheme is described in Figure 1. Specifically, we classified children into comparator groups as *definite pneumococcal pneumonia* (*S. pneumoniae* cultured from blood or pleural fluid), *probable pneumococcal pneumonia* (CRP concentration ≥60 mg/l and NP carriage of serotype 1 pneumococci), *probable bacterial pneumonia* (CRP concentration ≥60 mg/l only), *unknown pneumonia* (CRP concentration <60 mg/l only), *influenza/parainfluenza viral pneumonia* (CRP <60 mg/l and influenza/parainfluenza viruses detected by qPCR from NP specimen), *RSV pneumonia* (CRP <60 mg/l and RSV detected by qPCR from NP specimen), and *definite other bacterial pneumonia* (other bacterial pathogen cultured from blood or pleural fluid). These were ordered by hypothesized probability of pneumococcal infection from *definite pneumococcal pneumonia* to *definite other bacterial pneumonia* (left to right, Figure 1).

To assess the utility of acute IgG ALS to pneumococcal proteins to identify pneumococcal pneumonia we compounded *definite pneumococcal pneumonia* and *probable pneumococcal pneumonia* into a single category (*pneumococcal pneumonia*); and we combined *influenza/parainfluenza virus pneumonia, RSV pneumonia* and *definite other bacterial pneumonia* into a single category (*non-pneumococcal pneumonia*).

The classification scheme was modified from similar schemes for the development of novel diagnostic tests to discriminate between bacterial and viral infection (Herberg et al., 2016; Kaforou et al., 2017). Recent data have estimated a threshold to discriminate between bacterial and viral pneumonia at a CRP concentration of ~50–60 mg/l in children in South Asia and in low and middle-income countries in the PERCH study (Lubell et al., 2015; Higdon et al., 2017b). We chose NP carriage of pneumococcal serotype 1 (by culture) as a predictor of pneumococcal infection. The association of NP carriage of serotype 1 pneumococci in cases compared with controls, has been previously established in unvaccinated populations (Scott et al., 1996). NP carriage of serotype 1 was also associated with cases in comparison with controls in 938 children with pneumonia at Patan Hospital between 2014 and 2015 (prior to 10-valent PCV introduction) and 3,202 age-stratified control (non-pneumonia) children (odds ratio for case status of 5.0, 95% confidence interval, CI, 1.7–14.0; Figure S1). This was supported by unpublished data from childhood invasive pneumococcal disease at Patan Hospital from 2005 to 2016, where 58/124 (47%) isolated pneumococci were of serotype 1. Similar findings have been reported from other epidemiological settings (Scott et al., 1996; Johnson et al., 2010). We chose NP carriage (as detected by PCR) of influenza or parainfluenza virus, and RSV to represent viral pneumonia since carriage of these viruses was associated with radiographically-confirmed pneumonia in the PERCH study (Feikin et al., 2017a).

While we used NP carriage of serotype 1 pneumococci as a predictor of *pneumococcal pneumonia*, we also subsequently investigated for an association of NP carriage of pneumococci with increased IgG ALS to pneumococcal proteins. To avoid confounding with undetected pneumococcal infection, we limited this analysis to children with *non-pneumococcal pneumonia* (i.e., *definite other bacterial pneumonia, influenza/parainfluenza pneumonia* or *RSV pneumonia*).

### Statistical Methods

Continuous variables were compared using Student's *t*-test, following appropriate transformation (log transformation of age distribution; and taking the reciprocal of ALS concentrations to pneumococcal protein for the comparison of ALS concentrations to pneumococcal proteins between *probable bacterial pneumonia* and *unknown pneumonia*). Assay signals that were undetectable were assigned a value below the threshold of detection. Fisher exact tests, Wilcoxon rank sum tests, and χ^2^ test were used as indicated. Kruskal-Wallis tests were used to assess for confounding of acute IgG ALS to pneumococcal proteins with age, and length of illness. Formal statistical testing for an interaction between age (grouped, due to a non-normal distribution) and acute IgG ALS to pneumococcal proteins stratified by comparator group was not possible due to small numbers and tied ALS values. Analyses were pre-specified, with the exception of a *post-hoc* analysis of the effects of age on IgG ALS to pneumococcal proteins. We used best subsets regression analysis to investigate optimal combinations of pneumococcal protein antigens for IgG ALS to discriminate between children with *pneumococcal pneumonia* and *non-pneumococcal pneumonia* and assessed for multiple co-linearity.

## Results

### Characteristics of the Cohort

**Figure 2:**
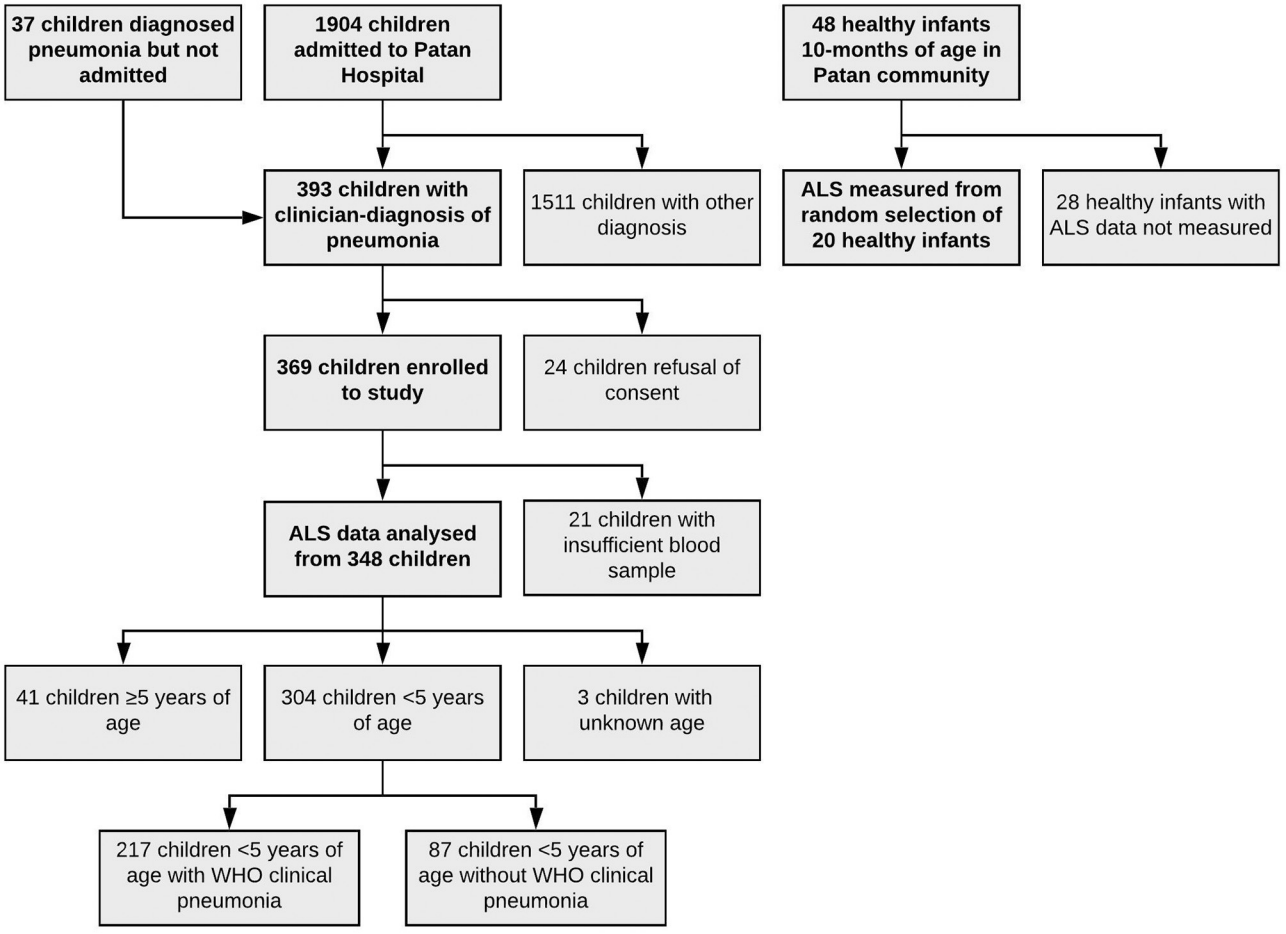
Flow diagram of enrolment to the study.

One thousand nine hundred four children were admitted to Patan Hospital during the study period. Of admitted children, 356 (19%) children had a clinician diagnosis of pneumonia, as had an additional 37 children who were diagnosed with pneumonia, but not admitted, totaling 393 children. 369 children were enrolled to the study, with data on ALS to pneumococcal proteins available on 348 of these children (Figure 2). Of 348 children, 122 (35%) had endpoint consolidation/effusions on chest radiograph, 32 (9%) had infiltrates, 178 had neither consolidation/effusions or infiltrates, 2 (<1%) were uninterpretable and 14 (4%) children did not have a chest radiograph. Of 304 children <5 years of age, 217 (71%) met the WHO criteria for pneumonia (fast breathing or chest indrawing) and 87 (29%) did not. In addition, 48 healthy infants (controls) aged 10 months of age were enrolled, on whom ALS was analyzed on a random subset of 20 infants. All healthy infant controls had received three doses of 10-valent PCV, with the most recent dose ≥28 days prior to enrolment to this study. The clinical characteristics of children with pneumonia are described in Table 1 by comparator group (and Table S1 by other classifications).

**Table 1:** Clinical characteristics of children of all ages with pneumonia enrolled to the study, by comparator group.

**Clinical characteristic**	***Pneumococcal pneumonia***				***Non-pneumococcal pneumonia***			
	***Definite pneumococcal pneumonia***	***Probable pneumococcal pneumonia***	***Probable bacterial pneumonia***	***Unknown***	***Influenza/para-influenza pneumonia***	***RSV pneumonia***	***Definite other bacterial pneumonia***	***p***
*n*	8	4	66	214	23	27	6^a^	
Age (years; median, IQR)	5.6 (4.2–8.1)	7.5 (5.8–8.3)	2.7 (1.7–5.1)	1.1 (0.6–2.0)	1.1 (0.7–2.5)	0.6 (0.3–1.5)	2.0 (0.7–7.4)	<0.001^b^
2–11 months	0	0	10 (15%)	93 (44%)	11 (48%)	19 (70%)	3 (50%)	
12–23 months	0	0	11 (17%)	64 (30%)	4 (17%)	3 (11%)	0	
24–59 months	3 (38%)	1 (25%)	27 (42%)	42 (20%)	7 (30%)	5 (19%)	1 (17%)	
≥5–14 years	5 (63%)	3 (75%)	17 (26%)	13 (6%)	1 (4%)	0	2 (33%)	
Female sex	1 (13%)	0	27 (41%)	86 (40%)	9 (39%)	15 (56%)	2 (33%)	0.27^c^
Length of illness (days; median, IQR)	2.5 (2–3)	3.5 (2.8–4.3)	4 (3–6)	3 (3–5.8)	5 (3–6.5)	4 (3–6.5)	6 (4.5–6.8)	0.32^b^
Prior antibiotic use	6 (75%)	2 (50%)	30 (45%)	94 (44%)	13 (56%)	11 (41%)	6 (100%)	0.25^c^
NP pneumococcal carriage	2 (25%)	4 (100%)	25 (38%)	59 (28%)	8 (35%)	4 (15%)	1 (17%)	0.02^c^
NP pneumococcal carriage (serotype 1)	1 (12.5%)	4 (100%)	0	1 (0.5%)	0	0	0	–
Endpoint consolidation	8 (100%)	3 (75%)	47 (71%)	51 (24%)	6 (26%)	4 (15%)	3 (50%)	–
CRP concentration (mg/l; median, IQR)	142 (56–183)	133 (97–171)	115 (82–183)	9.7 (2.7–22)	12.4 (5.6–19)	7.0 (1.5–23)	52 (14–113)	–
CRP concentration ≥60 mg/l	5 (63%)	4 (100%)	66 (100%)	0	0	0	3 (50%)	–
NP RSV carriage	0	0	3 (4.9%)	0	1 (4.3%)	27 (100%)	0	–
NP other viral carriage	0	0	4 (6.6%)	0	23 (100%)	0	0	–

a *Three Staphylococcus aureus, one each of Neisseria meningitidis, Pseudomonas spp., and Escherichia coli*.

b *Kruskal-Wallis test*.

c *Fisher exact test (simulated p-values). P-values were not calculated for variables that were entered into the classification scheme in Figure 1. Values are expressed as the percentage of n for each column (excepting continuous variables)*.

We classified eight children as *definite pneumococcal*, four children as *probable pneumococcal* (totaling 12 as *pneumococcal pneumonia*), 66 children as *probable bacterial*, 214 children as *unknown*, 23 children as *parainfluenza/influenza virus*, 27 children as *RSV*, and six children as *definite other bacterial pneumonia*. This totaled 56 children as *non-pneumococcal pneumonia*.

On comparison of children with *pneumococcal pneumonia* vs. children with *non-pneumococcal pneumonia*, there were significant differences in age (median 6.3 years, interquartile range 4.2–8.3 and 0.8 years, IQR 0.5–2.2, *t*-test following log transformation of age distribution, *p* < 0.001), sex (females 8 and 46%, Fisher exact test, *p* = 0.02), length of illness (3 days, IQR 2–3.3 and 4.5 days, IQR 3–7, Wilcoxon rank sum test, *p* < 0.001), and proportion with radiographic endpoint consolidation (92 and 23%, χ^2^ test, *p* < 0.001). The youngest child with *pneumococcal pneumonia* was 3.8 years of age.

### Diagnostic Accuracy of IgG ALS to Pneumococcal Proteins

IgG ALS to pneumococcal proteins was detected and quantifiable in 348 acute samples from children with pneumonia.

**Figure 3:**
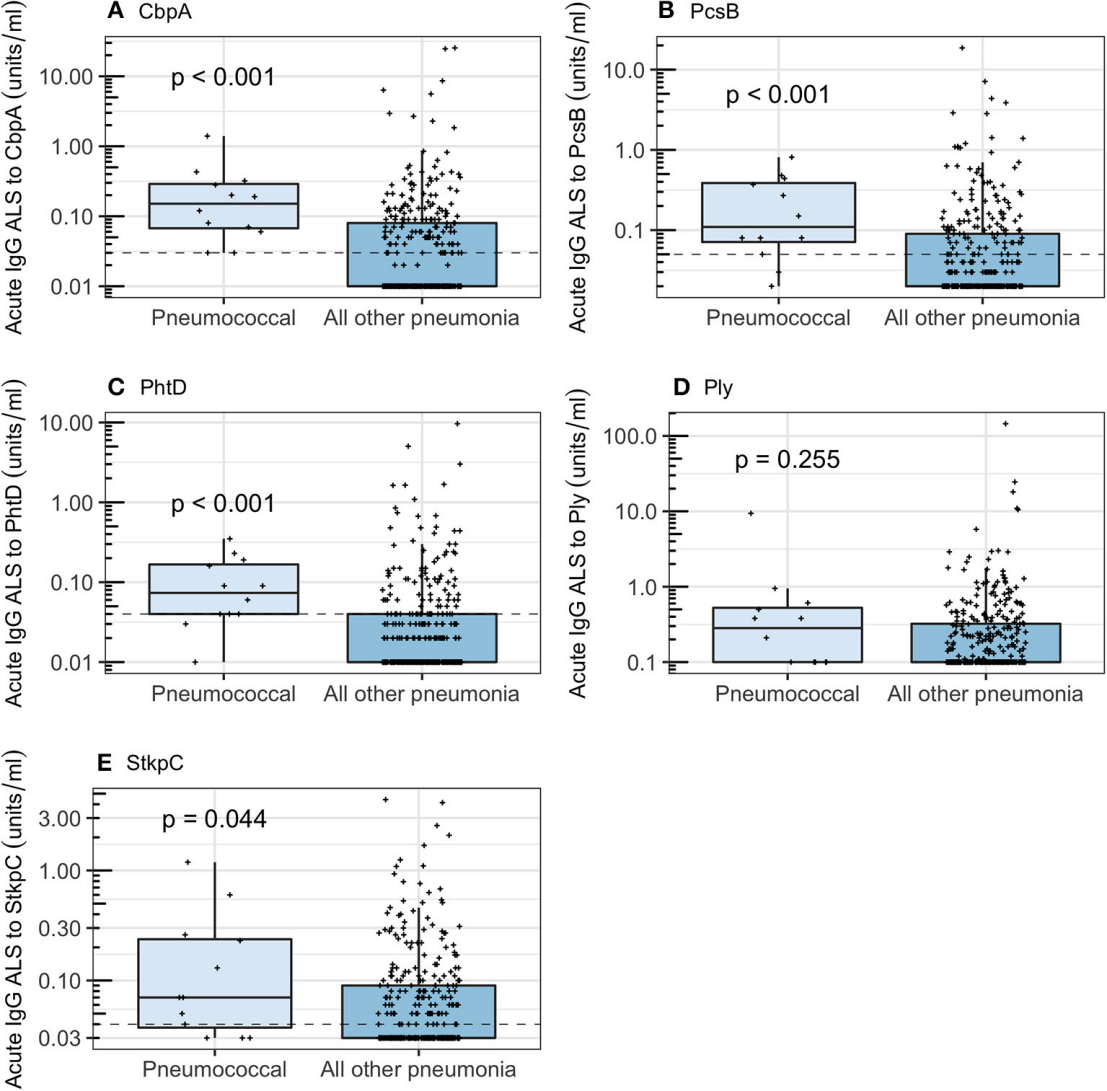
Acute IgG ALS to pneumococcal proteins by children with *pneumococcal pneumonia* and all other pneumonia in children enrolled into the study. Dashed horizontal lines represent thresholds derived from the Youden Index, *p*-values were derived from the Wilcoxon rank sum test. For all box and whisker plots: the solid line represents the median value, lower hinge 25th centile, upper hinge 75th centile, and whiskers represent 1.5 times the interquartile range. All data points have also been plotted. **(A)** Choline binding protein A (CbpA); **(B)** protein for cell wall separation of group B streptococci (PcsB); **(C)** pneumococcal histidine triad D (PhtD); **(D)** pneumolysin (Ply); **(E)** serine threonine kinase protein C (StkpC).

Acute IgG ALS was higher in children with *pneumococcal pneumonia* than children with all other pneumonia (including possible cases of undiagnosed pneumococcal pneumonia within the comparator groups *probable bacterial pneumonia, unknown, influenza/parainfluenza pneumonia, RSV pneumonia, definite other bacterial pneumonia*) for 4/5 pneumococcal proteins (Figure 3). Assay of acute IgG ALS to pneumococcal proteins discriminated between pneumococcal pneumonia and all other pneumonia with good accuracy for CbpA, PcsB and PhtD, but not Ply or StkpC: AUROC curve was 0.81 (95% CI 0.73–0.89) for CbpA, 0.77 (95% CI 0.65, 0.89) for PcsB, 0.78 (95% CI 0.67–0.89) for PhtD, 0.59 (95% CI 0.42–0.76) for Ply, and 0.66 (95% CI 0.50–0.81) for StkpC.

**Figure 4:**
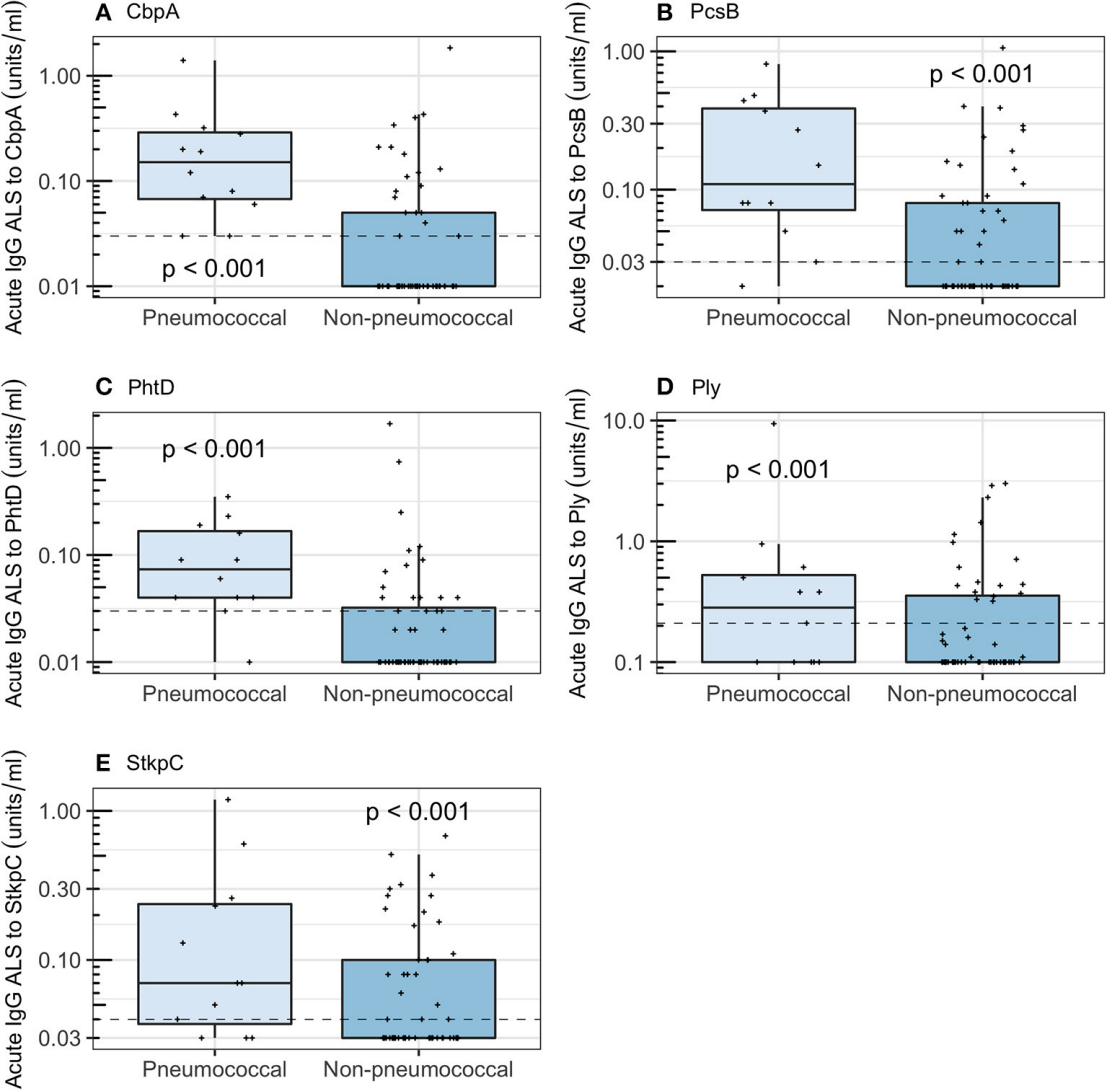
Acute IgG ALS to pneumococcal proteins by children with *pneumococcal pneumonia* and *non-pneumococcal pneumonia* in all children enrolled into the study. Dashed horizontal lines represent thresholds derived from the Youden Index, *p*-values were derived from the Wilcoxon rank sum test. **(A)** Choline binding protein A (CbpA); **(B)** protein for cell wall separation of group B streptococci (PcsB); **(C)** pneumococcal histidine triad D (PhtD); **(D)** pneumolysin (Ply); **(E)** serine threonine kinase protein C (StkpC).

Acute IgG ALS was higher in children with *pneumococcal pneumonia* than with *non-pneumococcal pneumonia* for all five pneumococcal proteins (Figure 4). Acute IgG ALS to pneumococcal proteins discriminated between *pneumococcal pneumonia* and *non-pneumococcal pneumonia* in children enrolled to the study with good sensitivity and specificity, with AUROC curve ranging from 0.60 (95% CI 0.42–0.79) for Ply, to 0.85 (95% CI 0.75–0.94) for CbpA, using thresholds derived from the Youden Index (Table 2). There was a high degree of co-linearity between acute IgG ALS to all five pneumococcal proteins measured with *pneumococcal pneumonia* (and *non-pneumococcal pneumonia*) in best subsets logistic regression analysis.

**Table 2:** Diagnostic accuracy, using thresholds derived from the Youden Index, for acute IgG ALS to pneumococcal proteins to discriminate between *pneumococcal pneumonia* and *non-pneumococcal pneumonia* in children with pneumonia in Nepal (all age groups).

***Pneumococcal pneumonia*** **and** ***non-pneumococcal pneumonia***					
	**CbpA**	**PcsB**	**PhtD**	**Ply**	**StkpC**
Cut-off value	0.03	0.03	0.03	0.21	0.04
Sensitivity	1.0 (0.73–1.0)	0.92 (0.62–1.0)	0.92 (0.62–1.0)	0.58 (0.28–0.85)	0.75 (0.43–0.95)
Specificity	0.66 (0.52–0.78)	0.57 (0.43–0.70)	0.68 (0.54–0.80)	0.70 (0.56–0.81)	0.57 (0.43–0.70)
AUROCC	0.85 (0.75–0.94)	0.79 (0.65–0.92)	0.82 (0.70–0.95)	0.60 (0.42–0.79)	0.66 (0.50–0.83)

*AUROCC, area under the receiver-operating characteristic curve*.

Discriminating between *pneumococcal pneumonia* patients and healthy controls, acute IgG ALS had an AUROC curve ranging from 0.68 for Ply (95% CI 0.49–0.87) to 0.98 for CbpA (95% CI 0.94–1.0; Figure S2, Table S2).

Among children with *pneumococcal pneumonia*, there was no significant increase in acute IgG ALS to any of the pneumococcal proteins measured with increasing length of illness (simple linear regression, *p* > 0.5 for all five proteins; Figure S3). There were too few female cases with *pneumococcal pneumonia* to investigate sex differences.

Among children with *non-pneumococcal pneumonia*, there was no significant difference in acute IgG ALS to pneumococcal proteins between children with viral pneumonia (influenza/parainfluenza, RSV) or definite other bacterial pneumonia (CbpA, *p* = 0.06; PcsB, *p* = 0.50; PhtD, *p* = 0.28; Ply, *p* = 0.92; StkpC, 0.93).

**Figure 5:**
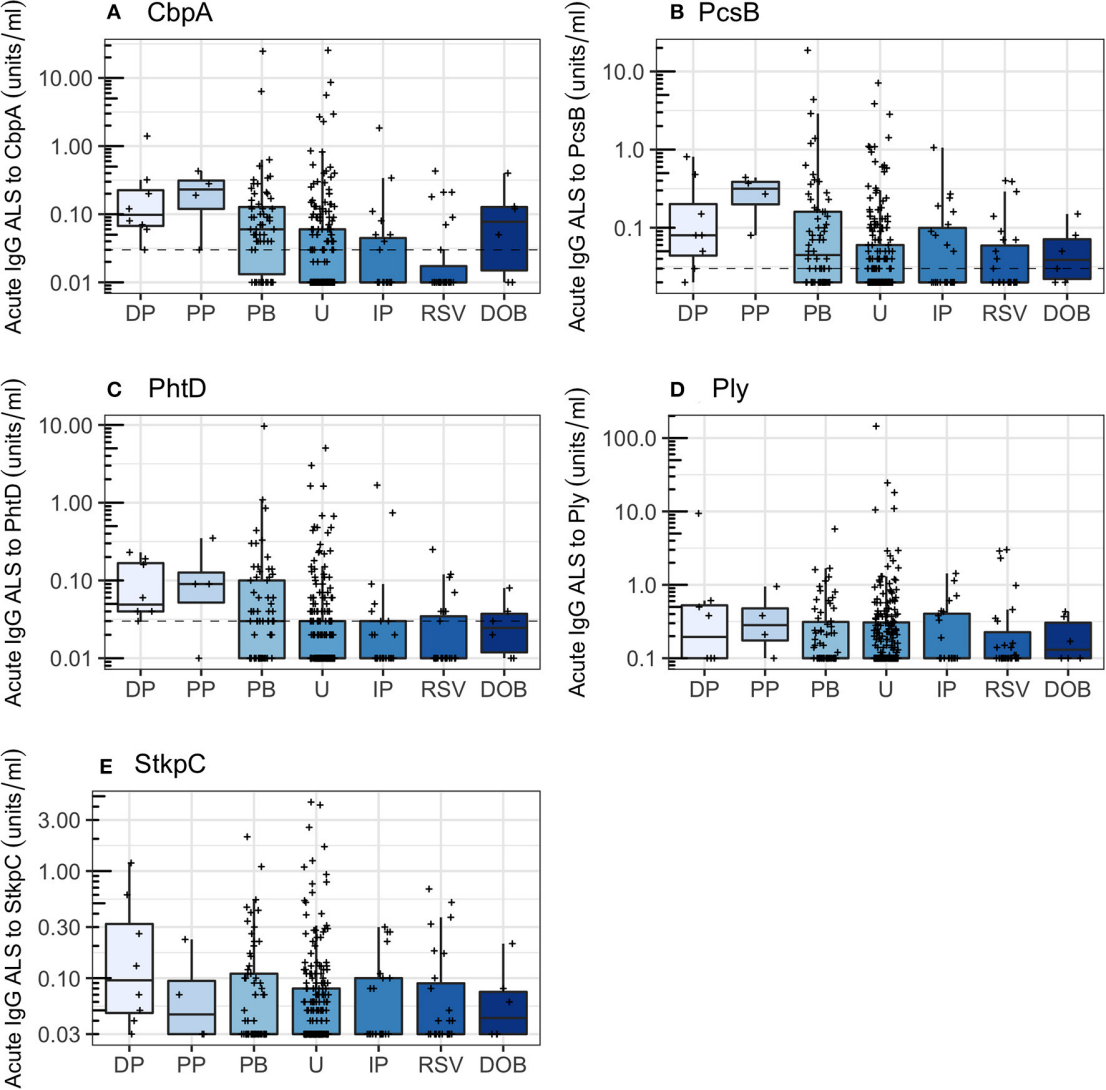
Acute IgG ALS to pneumococcal proteins by comparator group in all children enrolled into the study. Dotted horizontal lines represent thresholds derived from the Youden Index. DP, *definite pneumococcal*; PP, *probable pneumococcal*; PB, *probable bacterial*; U, *unknown*; IP *influenza/parainfluenza virus*; RSV, *respiratory syncytial virus*; DOB, *definite other bacterial pneumonia*. Dashed horizontal lines represent thresholds derived from the Youden Index. **(A)** Choline binding protein A (CbpA); **(B)** protein for cell wall separation of group B streptococci (PcsB); **(C)** pneumococcal histidine triad D (PhtD); **(D)** pneumolysin (Ply); **(E)** serine threonine kinase protein C (StkpC).

Children with *probable bacterial pneumonia* had higher concentrations of acute IgG ALS to CbpA, PcsB, PhtD, Ply, and StkpC than children with *unknown pneumonia* (Wilcoxon rank sum test, *p* < s0.001 for all). The proportion of children with acute IgG ALS concentrations greater than the threshold derived from ROC curve analysis differed significantly for CbpA (71% of *probable bacterial pneumonia* and 32% of *unknown pneumonia*, χ^2^ test, *p* < 0.001), PcsB (55 and 35%, *p* = 0.007), PhtD (45 and 25%, *p* = 0.002), but did not differ for Ply and StkpC (threshold lines not applicable to Figure 5).

### Sensitivity Analyses: Age Distribution

*Pneumococcal pneumonia* was only diagnosed in children ≥2 years of age. We therefore undertook a *post-hoc* analysis of acute IgG to pneumococcal proteins in children ≥2 years of age for the etiological diagnosis of pneumonia. Children ≥2 years of age known to have a qualitatively different humoral immune response to pneumococcal polysaccharide antigens, although not pneumococcal protein antigens, in comparison with young infants (Clutterbuck et al., 2007; Borges et al., 2016; Ramos-Sevillano et al., 2019). The clinical characteristics of children ≥2 years of age with *pneumococcal pneumonia* or *non-pneumococcal pneumonia* from the cohort are described in Table 3.

**Table 3:** Acute IgG ALS to pneumococcal proteins by children with *pneumococcal pneumonia* and *non-pneumococcal* pneumonia in children ≥2 years of age enrolled in the study.

**Clinical characteristic**	***Pneumococcal pneumonia***	***Non-pneumococcal pneumonia***	***p***
*n*	12	16	
Age (years; median, IQR)	6.3 (4.2–8.3)	3.2 (2.3–4.3)	<0.001^a^
24–59 months	4 (33%)	13 (81%)	–
≥5–14 years	8 (67%)	3 (19%)	–
Female sex	1 (8%)	11 (69%)	0.002^b^
Length of illness (days; median, range)	3 (2–3.3)	5.5 (3–7)	<0.001^c^
Prior antibiotic use	8 (67%)	9 (56%)	0.70^b^
NP pneumococcal carriage	6 (50%)	11 (69%)	0.54^c^
NP pneumococcal carriage (serotype 1)	5 (42%)	0	–
Invasive pneumococcal disease	8 (67%)	0	–
Other invasive bacterial disease^d^	0	3 (19%)	–
Endpoint consolidation	11 (92%)	7 (44%)	–
CRP (mg/l; median, IQR)	133 (63–183)	21 (14–50)	0.02^c^
CRP concentration ≥60 mg/l	9 (75%)	3 (19%)	–
NP RSV carriage	0	11 (31%)	–
NP other viral carriage	0	8 (50%)	–

*Values are expressed as a percentage for each column. p represents tests between the two groups*.

a *t-test following reciprocal transformation of age distribution for non-pneumococcal pneumonia*.

b *Fisher exact test*.

c *Wilcoxon rank sum test; χ^2^ test*.

d *Two Staphylococcus aureus, one Pseudomonas spp*.

**Figure 6:**
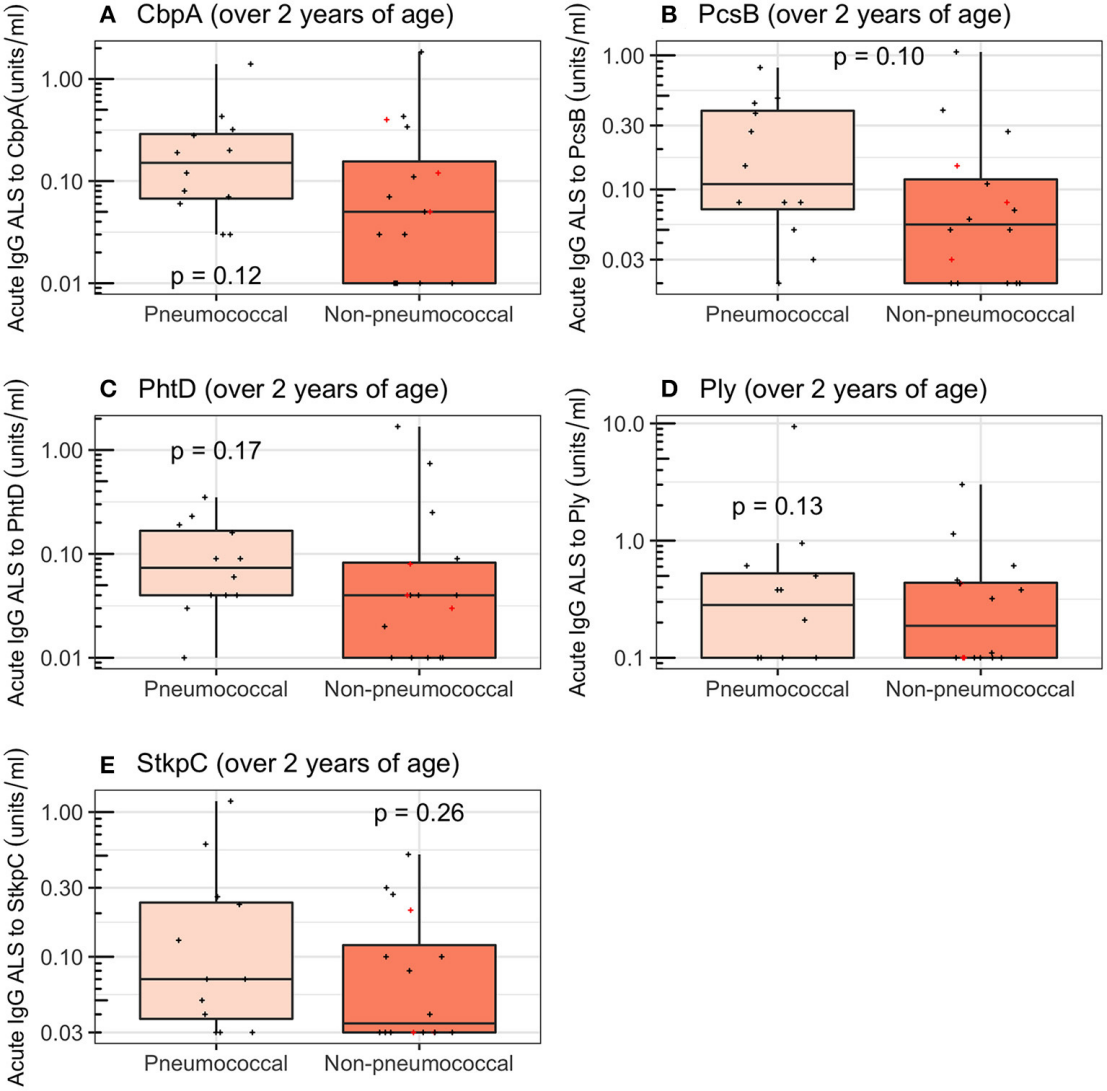
Acute IgG ALS to pneumococcal proteins in children with *pneumococcal pneumonia* and *non-pneumococcal pneumonia* ≥2 years of age enrolled into the study. Red crosses represent children with definite other bacterial pneumonia (two children with *Staphylococcus aureus*, and one child with *Pseudomonas* spp. isolated from blood). Dashed horizontal lines represent thresholds derived from the Youden Index, *p*-values were derived from the Wilcoxon rank sum test. **(A)** Choline binding protein A (CbpA); **(B)** protein for cell wall separation of group B streptococci (PcsB); **(C)** pneumococcal histidine triad D (PhtD); **(D)** pneumolysin (Ply); **(E)** serine threonine kinase protein C (StkpC).

Restricting the analysis to children ≥2 years of age reduced the ability to discriminate between *pneumococcal pneumonia* (12 children) and *non-pneumococcal pneumonia* (16 children) for all pneumococcal proteins to non-significance in this smaller number of samples (Wilcoxon rank sum tests; CbpA, *p* = 0.12; PcsB, *p* = 0.10; PhtD, *p* = 0.17; Ply, *p* = 0.13; StkpC, *p* = 0.26; Figure 6 and Table 4). A visual examination of data points did not suggest that children with *definite other bacterial pneumonia* had different acute IgG ALS to pneumococcal proteins than other children with *non-pneumococcal pneumonia* (red crosses and black crosses, Figure 6).

**Table 4:** Diagnostic accuracy, using thresholds derived from the Youden Index, for acute IgG ALS to pneumococcal proteins to discriminate between *pneumococcal pneumonia* and *non-pneumococcal pneumonia* in children ≥2 years of age with pneumonia in Nepal.

***Pneumococcal pneumonia*** **and** ***non-pneumococcal pneumonia*** **in children ≥2 years of age**					
	**CbpA**	**PcsB**	**PhtD**	**Ply**	**StkpC**
Cut-off value	0.06	0.08	0.03	0.50	0.04
Sensitivity	0.83 (0.52–0.98)	0.75 (0.43–0.95)	0.92 (0.61–1.00)	0.33 (0.10–0.65)	0.75 (0.43–0.95)
Specificity	0.56 (0.30–0.80)	0.63 (0.35–0.85)	0.38 (0.15–0.65)	0.81 (0.54–0.96)	0.50 (0.25–0.75)
AUROCC	0.67 (0.47–0.88)	0.69 (0.48–0.89)	0.65 (0.44–0.86)	0.53 (0.31–0.74)	0.61 (0.40–0.82)

*AUROCC, area under the receiver-operating characteristic curve*.

### Effects of Nasopharyngeal Carriage of Pneumococci

**Figure 7:**
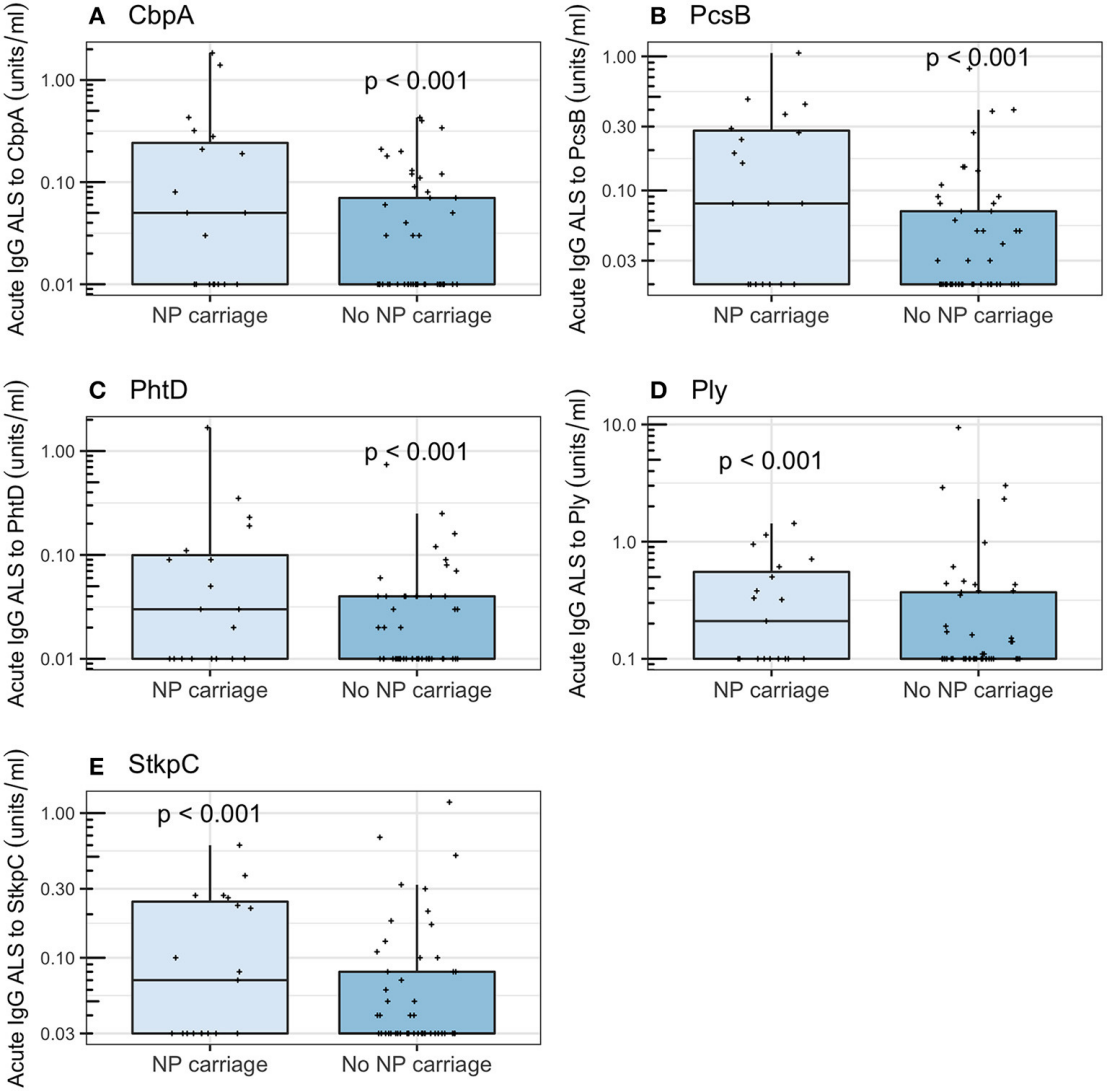
Acute IgG ALS to pneumococcal proteins in children with *non-pneumococcal pneumonia* and NP carriage of pneumococci and without NP carriage of pneumococci enrolled into the study (all age groups). *P*-values were derived from the Wilcoxon rank sum test. **(A)** Choline binding protein A (CbpA); **(B)** protein for cell wall separation of group B streptococci (PcsB); **(C)** pneumococcal histidine triad D (PhtD); **(D)** pneumolysin (Ply); **(E)** serine threonine kinase protein C (StkpC).

Among all children enrolled, those with NP carriage of pneumococci had higher acute IgG ALS than those without NP carriage of pneumococci (Wilcoxon rank sum tests, *p* < 0.001 for all five pneumococcal proteins). Among children with *non-pneumococcal pneumonia* (i.e., not “confounded” by *definite pneumococcal* or *probable pneumococcal* or *probable bacterial* or *unknown pneumonia*), those with NP carriage of pneumococci had higher acute IgG ALS to all five pneumococcal proteins than those without NP carriage (Wilcoxon rank sum tests; CbpA, *p* < 0.001; PcsB, *p* < 0.001; PhtD, *p* < 0.001; Ply, *p* < 0.001; StkpC, *p* < 0.001; Figure 7). Among children ≥2 years of age with *non-pneumococcal pneumonia*, there were no significant differences in acute IgG ALS to any pneumococcal protein detected between those with (*n* = 19) and without (*n* = 49) NP carriage of pneumococci (Wilcoxon rank sum tests, *p* > 0.5 for all comparisons, Figure S4). Stratification of this age group to those who had not received antibiotics prior to NP sampling did not significantly affect these results.

### Final Analysis

**Figure 8:**
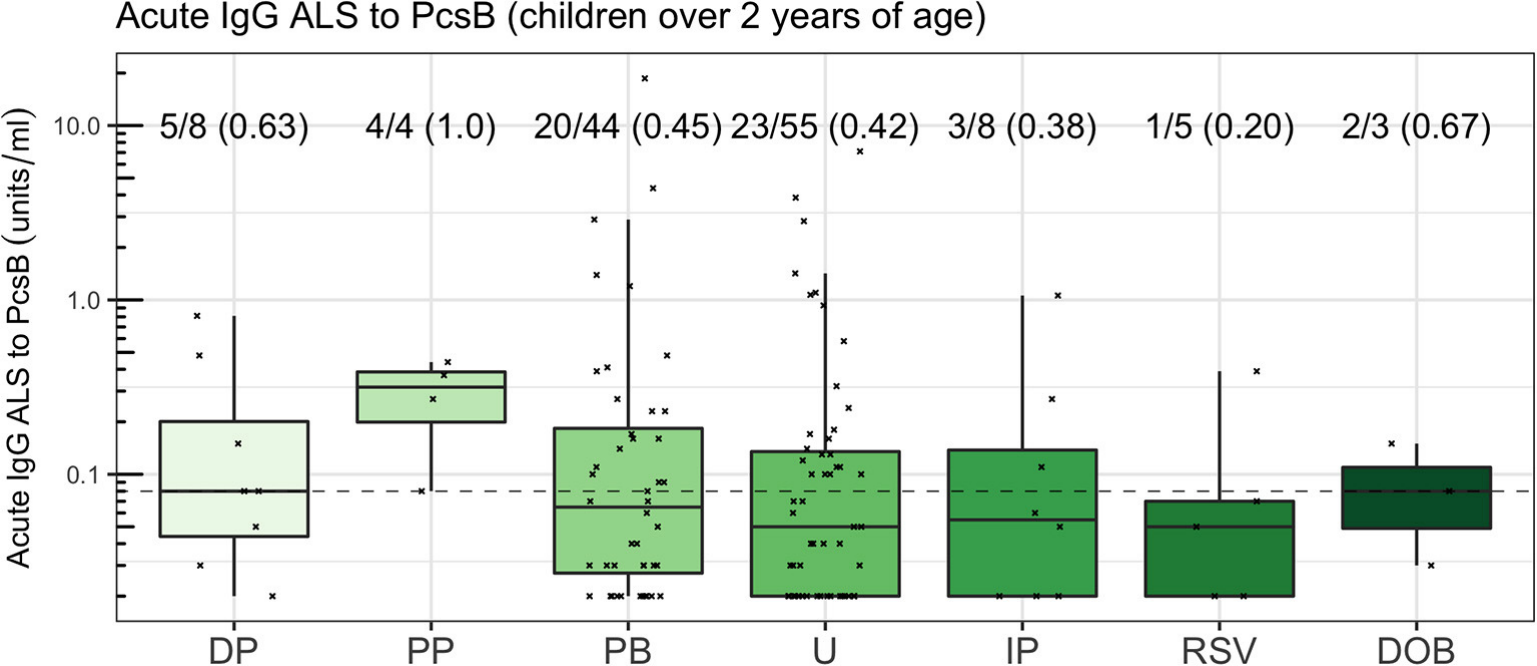
Acute IgG ALS to PcsB by comparator group in children ≥2 years of age. The dotted horizontal line represents a threshold of 0.08 units/ml, the threshold derived from the Youden Index. The proportion of children with acute IgG ALS to PcsB concentration greater than, or equal to, the threshold derived from ROC curve analysis to discriminate *pneumococcal pneumonia* from *non-pneumococcal pneumonia* is annotated.

We used acute IgG ALS to PcsB as the most parsimonious approach to investigate acute IgG to pneumococcal proteins in children ≥2 years of age across the cohort. The number of children (proportion) that had acute IgG ALS greater than, or equal to, the optimum threshold to discriminate between *pneumococcal pneumonia* and *non-pneumococcal pneumonia* was: *definite pneumococcal pneumonia* 5/8 (0.63), *probable pneumococcal pneumonia* 4/4 (1.0), *probable bacterial pneumonia* 20/44 (0.45), *unknown pneumonia* 23/55 (0.42), *influenza/parainfluenza pneumonia* 3/8 (0.38), *RSV pneumonia* 1/5 (0.2), *definite other bacterial pneumonia* 2/3 (0.67; Figure 8).

## Discussion

The development of novel diagnostic tests for the etiology of pneumonia is partly driven by the need to assess the impact of vaccination strategies on the total, and pathogen-specific burden of pneumonia in diverse settings. We have chosen to focus our work on pneumococcal pneumonia, during the introduction of 10-valent PCV to the infant immunization schedule in Nepal.

Currently available diagnostic tests for pneumonia etiology lack sensitivity or specificity in children. Culture of pneumococci from blood or pleural fluid from children with pneumonia is presumed to be highly specific for pneumococcal pneumonia but lacks sensitivity (O'Brien et al., 2009; Wu et al., 2016). Theoretically, sampling infected tissue (the lung) might improve sensitivity. A review of studies using lung biopsy followed by culture for the diagnosis of pneumonia etiology identified an increase in yield of bacterial pathogens from 14 to 47% of children tested (Ideh et al., 2011). However, lung biopsy requires peripheral radiographic consolidation, and was therefore not possible in approximately three quarters of patients with clinically severe pneumonia in a recent prospective cohort (Howie et al., 2014). This, in addition to safety concerns, has prevented lung biopsy for diagnostic sampling from becoming a widespread technique (Ideh et al., 2011). Interpretation of samples from the nasopharynx of children is limited by poor specificity, with many pathogens detected at similar prevalence in children with pneumonia and in community controls (Higdon et al., 2017a). Culture or molecular detection of pathogens from of broncho-alveolar lavage samples is only possible on samples from children receiving mechanical ventilation.

We hypothesized that analysis of the immune response to pneumococci may be useful as a diagnostic approach to pneumonia etiology in children. We evaluated a new diagnostic strategy, assay of ALS, based on quantification of antibodies against pneumococcal proteins that are spontaneously secreted by transiently circulating lymphocytes from children with pneumonia. We were able to detect, and quantify, IgG ALS to five specific pneumococcal proteins (CbpA, PcsB, PhtD, Ply, and StkpC) from children with pneumonia using a multiplexed immunoassay (FMIA). Concentrations of ALS IgG to these antigens were significantly higher in supernatants from PBMCs obtained from children with *pneumococcal pneumonia* than from children with *non-pneumococcal pneumonia*. For children of all ages, acute IgG ALS to the best-performing antigen (CbpA) appeared highly sensitive, and moderately specific, with an AUROC curve of 0.85, for the discrimination of *pneumococcal pneumonia* from *non-pneumococcal pneumonia*. This is considerably higher than previously reported approaches, including quantification of pneumococcal DNA in blood, or in NP specimens by qPCR (Baggett et al., 2017; Deloria Knoll et al., 2017), to discriminate between children with microbiologically confirmed pneumococcal pneumonia and non-pneumococcal pneumonia, or between pneumococcal pneumonia and healthy controls.

Despite this reasonable performance, we observed a statistically significant association between acute IgG ALS and age, independent of diagnostic comparator group. The limited overlap in ages between children with *pneumococcal pneumonia* and *non-pneumococcal pneumonia* made adjustment for this important confounding variable infeasible. The age distribution of children with *pneumococcal pneumonia* in this study was consistent with unpublished data from long-term surveillance of childhood invasive pneumococcal disease at Patan Hospital, but is in contrast to other sites in South Asia where 50% or more of confirmed pneumococcal pneumonia is detected in infants (Baqui et al., 2007; Arifeen et al., 2009; Saha et al., 2015; Manoharan et al., 2017).

We therefore undertook *post-hoc* analysis in children ≥2 years of age. In this age group, ALS assay to the best-performing antigen (PcsB) was unable to discriminate between children with *pneumococcal pneumonia* and *non-pneumococcal pneumonia* with an AUROC curve of 0.69 (with wide confidence intervals in these data). Our experience suggests that this is insufficiently sensitive or specific for clinical use. Assay of acute IgG ALS may be informative for the etiological diagnosis of childhood pneumonia in epidemiological studies, but future studies will need careful accounting for age between comparator groups.

We also described the effect of NP carriage of pneumococci on acute IgG ALS in children with *non-pneumococcal pneumonia*. Previous work has shown specific IgG and IgA responses can be induced through stimulation of *ex vivo* child adenoidal mononuclear cells with pneumococcal protein antigens. These ALS responses were found to be positively associated with age and serum antibody concentrations, and were higher from cells from adenoids colonized by pneumococci (Zhang et al., 2002, 2006). Our data suggest that NP carriage of pneumococci in pneumonia—without apparent pneumococcal disease (Palkola et al., 2012, 2016)—also induces IgG secretion from antibody-secreting cells, thus confounding the utility of an ALS-based diagnostic approach.

Strengths of this study include the unselected cohort, and the breadth of clinical and microbiological data used to classify the likely etiology of pneumonia by comparator group. In April and May 2015 central Nepal (including the Kathmandu valley) experienced earthquakes that led to the deaths of ~8,000 people in the central region of Nepal, and large population movements (Hall et al., 2017). In addition, a severe fuel shortage and increased construction from September 2015 to February 2016 may have contributed to severe pollution in the Kathmandu Valley through the increased burning of biofuels (Budhathoki and Gelband, 2016). Both earthquakes and increased pollution may have affected pneumococcal pneumonia incidence at Patan Hospital during the study period.

Despite the lack of additional selection, our cohort was enriched for invasive pneumococcal disease (IPD) in comparison to similar cohorts within South Asia. Our cohort included 8 children bacteremic for the pneumococcus from 369 children (2.2%). In comparison, separate studies of pneumonia in rural Bangladesh [7 cases of IPD from 840 children, 0.8% (Baqui et al., 2007)], severe febrile illness [25 cases of IPD from 6,925 children, 0.4% (Arifeen et al., 2009)], and 220 cases of IPD from a meta-analysis of 26,258 blood cultures from children (0.1%) across south-east Asia between 1990 and 2010 (Deen et al., 2012), were identified. The recently published PERCH study isolated the pneumococcus in 19 cases from 4,232 children (0.4%) with pneumonia (O'Brien et al., 2019). Our cohort had a similar prevalence of IPD as among rural Gambian or rural Kenyan children with pneumonia [2.5% (Cutts et al., 2005) and 1.7%, respectively (Berkley et al., 2005)] prior to introduction of PCV.

Any test for pneumonia etiology should identify children in whom there is pneumococcal infection, but no bacteremia. For this reason, and to increase the number of children available for comparator standards, we defined four children with pneumonia, high CRP and NP carriage of serotype 1 as *pneumococcal pneumonia* (Figure 1). All of these children also had consolidation on chest radiograph. We chose NP carriage of serotype 1 due to its high odds ratio for carriage in pneumonia vs. community controls (Figure S1) in our unpublished data from Kathmandu, and in published data from Israel (Greenberg et al., 2011), the UK, and South America (Scott et al., 1996). Of 128 children with IPD admitted to Patan Hospital between 2005 and 2016, 58 (45%) had serotype 1 pneumococci identified. We therefore believe that class of children with NP carriage of serotype 1 and high CRP as pneumococcal pneumonia has biological relevance, and internal and external validity.

We focused our analysis on comparisons between children with *pneumococcal pneumonia* and *non-pneumococcal pneumonia*, rather than on discriminating between children with *pneumococcal pneumonia* and age-matched community controls. This substantially reduced the measured diagnostic accuracy of acute IgG ALS to pneumococcal proteins that we present in the main text (in this study: optimum antigen AUROC curve 0.98 for *pneumococcal pneumonia* and healthy controls; 0.84 for *pneumococcal pneumonia* and *non-pneumococcal pneumonia* in all ages; 0.69 for *pneumococcal pneumonia* and *non-pneumococcal pneumonia* in children ≥2 years of age). However, testing acute IgG ALS to pneumococcal proteins for the diagnosis of pneumococcal pneumonia, in the context of an unselected cohort of pneumonia patients (as presented here), rather than in healthy controls gives a meaningful representation of accuracy in the context in which the test would be potentially implemented.

Assay of ALS for the etiological diagnosis of pneumonia might be optimized to yield more accurate diagnostic information. Reviews of B cell responses to vaccination (Mitchell et al., 2014) and to infection (Carter et al., 2017) suggest that the optimum time for sampling transiently circulating plasmablasts from peripheral blood is 7–10 days following the onset of illness. There are difficulties defining length of illness in children with pneumonia. Nevertheless, the median length of illness prior to blood sampling in our study was 4 days. A delay in sampling until 7 days of illness may improve test specificity. An additional method to improve specificity of the ALS assay would be to sort recently activated plasmablasts for incubation using flow cytometry. However, separation, washing, and incubation of PBMCs to generate and store ALS requires reagents (Ficoll-paque) and equipment (centrifuges, incubators with CO_2_, and −80°C freezers) that are not readily available in routine microbiology laboratories. The use of flow cytometry would limit the potential availability of this test further. Finally, although we described a high degree of co-linearity in acute IgG ALS to the pneumococcal proteins measured, a larger array of pneumococcal antigens might be considered to optimize ALS cognate antigens using variable selection methods (Zou and Hastie, 2005; Darton et al., 2017a). Quantification of ALS to pneumococcal capsular polysaccharides may also enable serotype-specific measure of pneumococcal pneumonia burden to inform vaccination strategies (Tuerlinckx et al., 2013).

A major limitation of any study of pneumonia etiology is the lack of a “gold standard.” Blood culture is presumed to be specific, but insensitive, for bacterial pneumonia—a “silver standard” (Wu et al., 2016). We used a series of comparator groups (“standards”) against which to test the ALS assay. In this cohort, the low prevalence and different age distribution of confirmed pneumococcal pneumonia cases, in comparison to other pneumonia cases, limited our measures of the diagnostic accuracy of ALS between comparator groups. Assay of ALS was also confounded by the association of acute IgG ALS with NP carriage of pneumococci. Diagnostic accuracy of ALS assay may be better in studies of pneumonia of other etiology (notably RSV pneumonia), where reasonably accurate diagnostic tests already exist with a high positive and negative predictive value for disease, as a point of comparison.

Despite the high prevalence of pneumococcal pneumonia in the cohort relative to other studies (Baqui et al., 2007; Arifeen et al., 2009; Deen et al., 2012; O'Brien et al., 2019), the majority of children in the study (81%) were designated as *probable bacterial pneumonia* or *unknown pneumonia*. In a cohort that were largely unvaccinated with PCV, only 8 children had invasive pneumococcal disease, with a further 4 children with *probable pneumococcal pneumonia*. Many comparisons were therefore underpowered. Although this limits the ability to fully assess assay of IgG ALS for the diagnosis of pneumococcal pneumonia, at a minimum, a clinically useful test should be “positive” in cases of IPD (the silver standard). Our data show that this was not the case, with low IgG ALS assay in 3 of 8 children with pneumococcal bacteremia (Figure 8).

In summary, we detected spontaneously secreted antibodies to pneumococcal proteins from PBMCs isolated from a proportion of children with pneumonia in Nepal using the ALS assay. Concentrations of IgG ALS to the pneumococcal proteins CbpA, PcsB and PhtD were higher in children with *pneumococcal pneumonia* than *non-pneumococcal pneumonia*, with good ability to discriminate between groups. However, these results were confounded by different age distributions of children with *pneumococcal pneumonia* and *non-pneumococcal pneumonia*. Assay of ALS to pneumococcal proteins did not discriminate between these groups when stratified by ≥2 years of age. Our data suggest that assay of IgG ALS to pneumococcal proteins is not sufficiently accurate as a diagnostic test for clinical utility. Alternative new diagnostic tests for the cause of childhood pneumonia should be sought.

## Data Availability Statement

The datasets generated for this study are available on request to the corresponding author.

## Ethics Statement

The studies involving human participants were reviewed and approved by Nepal Health Research Council and the Oxford Tropical Research Ethics Committee. Written informed consent to participate in this study was provided by the participants' legal guardian/next of kin.

## Author Contributions

MC, CJ, DM, AP, DK, and SS designed the study and analysis. MC, PG, SR, MG, ST, SK, IA, GS, MCG, KP, BK, and AM performed the studies and collected sample material. MC, PG, SR, RK, NE, and MM performed the assays. MC, JK, MV, KO'B, BW, AP, and DK performed the analyses. MC, DK, and AP wrote the manuscript to which all authors had significant input. MC, DK, JK, and AP acquired the funding directly for this study. This study was also supported by the PneumoNepal Study Group.

### Conflict of Interest

The authors declare that the research was conducted in the absence of any commercial or financial relationships that could be construed as a potential conflict of interest.

## Additional PneumoNepal Study Group members

Kate Park, Matthew Smedley, Puja Amatya, Ruby Basi, Jennifer Moïsi, Maria Deloria Knoll, Brad Gessner.

## Supplementary Material

Figure S1 Odds ratios for nasopharyngeal carriage of pneumococcal serotypes contained within 13-valent PCV and non-typeable (NT) pneumococci in pneumonia and community control children, adjusted for age and sex, prior to introduction of the vaccine into the Kathmandu valley. Error bars show 95% confidence intervals.

Figure S2 Acute IgG ALS to pneumococcal proteins by children with pneumococcal pneumonia and healthy infant controls. Dashed horizontal lines represent represent thresholds derived from the Youden Index, *p*-values were derived from the Wilcoxon rank sum test. For all box and whisker plots: the solid line represents the median value, lower hinge 25th centile, upper hinge 75th centile, and whiskers represent 1.5 times the interquartile range. All data points have also been plotted.

Figure S3 Linear regression analysis of length of illness with acute IgG ALS to pneumococcal proteins (grey colouring represents 95% confidence intervals about the regression line; *p*-value > 0.5 for all proteins).

Figure S4 Acute IgG ALS to pneumococcal proteins in children with non-pneumococcal pneumonia by nasopharyngeal carriage of pneumococci.

## References

[B1] Andrade D. C. Borges I. C. Ivaska L. Peltola V. Meinke A. Barral A.. (2016). Serological diagnosis of pneumococcal infection in children with pneumonia using protein antigens: a study of cut-offs with positive and negative controls. J. Immunol. Methods 433, 31–37. 10.1016/j.jim.2016.02.021 26928648

[B2] Andrade D. C. Borges I. C. Laitinen H. Ekström N. Adrian P. V. Meinke A.. (2014). A fluorescent multiplexed bead-based immunoassay (FMIA) for quantitation of IgG against *Streptococcus pneumoniae, Haemophilus influenzae* and *Moraxella catarrhalis* protein antigens. J. Immunol. Methods 405, 130–143. 10.1016/j.jim.2014.02.002 24530690

[B3] Arifeen S. E. Saha S. K. Rahman S. Rahman K. M. Rahman S. M. Bari S.. (2009). Invasive pneumococcal disease among children in rural Bangladesh: results from a population-based surveillance. Clin. Infect. Dis. 48, S103–S113. 10.1086/596543 19191605

[B4] Baggett H. C. Watson N. L. Deloria Knoll M. Brooks W. A. Feikin D. R. Hammitt L. L.. (2017). Density of upper respiratory colonization with *Streptococcus pneumoniae* and its role in the diagnosis of pneumococcal pneumonia among children aged <5 years in the PERCH study. Clin. Infect. Dis. 64, S317–S327. 10.1093/cid/cix100 28575365 PMC5850437

[B5] Baqui A. H. Rahman M. Zaman K. El Arifeen S. Chowdhury H. R. Begum N.. (2007). A population-based study of hospital admission incidence rate and bacterial aetiology of acute lower respiratory infections in children aged less than five years in Bangladesh. J. Health Popul. Nutr. 25, 179–188. Available online at: https://www.ncbi.nlm.nih.gov/pmc/articles/PMC2754000/17985819 PMC2754000

[B6] Berkley J. A. Lowe B. S. Mwangi I. Williams T. Bauni E. Mwarumba S.. (2005). Bacteremia among children admitted to a rural hospital in Kenya. N. Engl. J. Med. 352, 39–47. 10.1056/NEJMoa040275 15635111

[B7] Borges I. C. Andrade D. C. Cardoso M. R. Toppari J. Vähä-Mäkilä M. Ilonen J.. (2016). Natural development of antibodies against *Streptococcus pneumoniae, Haemophilus influenzae*, and *Moraxella catarrhalis* protein antigens during the first 13 years of life. Clin. Vaccine Immunol. 23, 878–883. 10.1128/CVI.00341-16 27581439 PMC5098021

[B8] Budhathoki S. S. Gelband H. (2016). Manmade earthquake: the hidden health effects of a blockade-induced fuel crisis in Nepal. BMJ Glob. Health 1:e000116. 10.1136/bmjgh-2016-000116 28588948 PMC5321345

[B9] Carter M. J. Mitchell R. M. Meyer Sauteur P. M. Kelly D. F. Trück J. (2017). The antibody-secreting cell response to infection: kinetics and clinical applications. Front. Immunol. 8:630. 10.3389/fimmu.2017.00630 28620385 PMC5451496

[B10] Chang H. S. Sack D. A. (2001). Development of a novel *in vitro* assay (ALS assay) for evaluation of vaccine-induced antibody secretion from circulating mucosal lymphocytes. Clin. Diagn. Lab. Immunol. 8, 482–488. 10.1128/CDLI.8.3.482-488.2001 11329444 PMC96087

[B11] Cherian T. Mulholland E. K. Carlin J. B. Ostensen H. Amin R. de Campo M.. (2005). Standardized interpretation of paediatric chest radiographs for the diagnosis of pneumonia in epidemiological studies. Bull WHO 83, 353–359. Available online at: https://www.ncbi.nlm.nih.gov/pmc/articles/PMC2626240/15976876 PMC2626240

[B12] Clutterbuck E. A. Oh S. Hamaluba M. Westcar S. Beverley P. C. L. Pollard A. J. (2007). Serotype-specific and age-dependent generation of pneumococcal polysaccharide-specific memory B-cell and antibody responses to immunization with a pneumococcal conjugate vaccine. Clin. Vaccine Immunol. 15, 182–193. 10.1128/CVI.00336-07 18032593 PMC2238039

[B13] Cutts F. T. Zaman S. M. Enwere G. Jaffar S. Levine O. S. Okoko J. B.. (2005). Efficacy of nine-valent pneumococcal conjugate vaccine against pneumonia and invasive pneumococcal disease in The Gambia: randomised, double-blind, placebo-controlled trial. Lancet 365, 1139–1146. 10.1016/S0140-6736(05)71876-6 15794968

[B14] Darton T. C. Baker S. Randall A. Dongol S. Karkey A. Voysey M.. (2017a). Identification of novel serodiagnostic signatures of typhoid fever using a Salmonella proteome array. Front. Microbiol. 8:1794. 10.3389/fmicb.2017.01794 28970824 PMC5609549

[B15] Darton T. C. Jones C. Dongol S. Voysey M. Blohmke C. J. Shrestha R.. (2017b). Assessment and translation of the antibody-in-lymphocyte supernatant (ALS) assay to improve the diagnosis of enteric fever in two controlled human infection models and an endemic area of Nepal. Front. Microbiol. 8:2031. 10.3389/fmicb.2017.02031 29109704 PMC5660281

[B16] Deen J. von Seidlein L. Andersen F. Elle N. White N. J. Lubell Y. (2012). Community-acquired bacterial bloodstream infections in developing countries in south and southeast Asia: a systematic review. Lancet Infect. Dis. 12, 480–487. 10.1016/S1473-3099(12)70028-2 22632186

[B17] Deloria Knoll M. Morpeth S. C. Scott J. A. G. Watson N. L. Park D. E. Baggett H. C.. (2017). Evaluation of pneumococcal load in blood by polymerase chain reaction for the diagnosis of pneumococcal pneumonia in young children in the PERCH study. Clin. Infect. Dis. 64, S357–S367. 10.1093/cid/cix101 28575374 PMC5447847

[B18] Driscoll A. J. Deloria Knoll M. Hammitt L. L. Baggett H. C. Brooks W. A. Feikin D. R.. (2017). The effect of antibiotic exposure and specimen volume on the detection of bacterial pathogens in children with pneumonia. Clin. Infect. Dis. 64, S368–S377. 10.1093/cid/cix149 28575366 PMC5447850

[B19] Feikin D. R. Fu W. Park D. E. Shi Q. Higdon M. M. Baggett H. C.. (2017a). Is higher viral load in the upper respiratory tract associated with severe pneumonia? Findings from the PERCH study. Clin. Infect. Dis. 64, S337–S346. 10.1093/cid/cix148 28575373 PMC5447843

[B20] Feikin D. R. Hammitt L. L. Murdoch D. R. O'Brien K. L. Scott J. A. G. (2017b). The enduring challenge of determining pneumonia etiology in children: considerations for future research priorities. Clin. Infect. Dis. 64, S188–S196. 10.1093/cid/cix143 28575369 PMC5447852

[B21] Feikin D. R. Scott J. A. G. Gessner B. D. (2014). Use of vaccines as probes to define disease burden. Lancet 383, 1762–1770. 10.1016/S0140-6736(13)61682-7 24553294 PMC4682543

[B22] Greenberg D. Givon-Lavi N. Newman N. Bar-Ziv J. Dagan R. (2011). Nasopharyngeal carriage of individual *Streptococcus pneumoniae* serotypes during pediatric pneumonia as a means to estimate serotype disease potential. Pediatr. Infect. Dis. J. 30, 227–233. 10.1097/INF.0b013e3181f87802 20861756

[B23] Hall M. L. Lee A. C. Cartwright C. Marahatta S. Karki J. Simkhada P. (2017). The 2015 Nepal earthquake disaster: lessons learned one year on. Public Health 145, 39–44. 10.1016/j.puhe.2016.12.031 28359388

[B24] Herberg J. A. Kaforou M. Wright V. J. Shailes H. Eleftherohorinou H. Hoggart C. J.. (2016). Diagnostic test accuracy of a 2-transcript host RNA signature for discriminating bacterial vs viral infection in febrile children. JAMA 316, 835–845. 10.1001/jama.2016.11236 27552617 PMC5997174

[B25] Higdon M. M. Hammitt L. L. Deloria Knoll M. Baggett H. C. Brooks W. A. Howie S. R. C.. (2017a). Should controls with respiratory symptoms be excluded from case-control studies of pneumonia etiology? Reflections from the PERCH study. Clin. Infect. Dis. 64, S205–S212. 10.1093/cid/cix076 28575354 PMC5447853

[B26] Higdon M. M. Le T. O'Brien K. L. Murdoch D. R Prosperi C Baggett H. C. (2017b). Association of C-reactive protein with bacterial and respiratory syncytial virus-associated pneumonia among children aged <5 years in the PERCH study. Clin. Infect. Dis. 64, S378–S386. 10.1093/cid/cix150 28575375 PMC5447856

[B27] Howie S. R. Morris G. A. Tokarz R. Ebruke B. E. Machuka E. M. Ideh R. C.. (2014). Etiology of severe childhood pneumonia in the Gambia, West Africa, determined by conventional and molecular microbiological analyses of lung and pleural aspirate samples. Clin. Infect. Dis. 59, 682–685. 10.1093/cid/ciu384 24867789 PMC4130311

[B28] Ideh R. C. Howie S. R. Ebruke B. Secka O. Greenwood B. M. Adegbola R. A.. (2011). Transthoracic lung aspiration for the aetiological diagnosis of pneumonia: 25 years of experience from The Gambia. Int. J. Tuberc. Lung Dis. 15, 729–735. 10.5588/ijtld.10.0468 21477423

[B29] Johnson H. L. Deloria-Knoll M. Levine O. S. Stoszek S. K. Freimanis Hance L. Reithinger R.. (2010). Systematic evaluation of serotypes causing invasive pneumococcal disease among children under five: the pneumococcal global serotype project. PLoS Med. 7:e1000348. 10.1371/journal.pmed.1000348 20957191 PMC2950132

[B30] Kaforou M. Herberg J. A. Wright V. J. Coin L. J. Levin M. (2017). Diagnosis of bacterial infection using a 2-transcript host RNA signature in febrile infants 60 days or younger. JAMA 317, 1577–1578. 10.1001/jama.2017.1365 28418473

[B31] Liu L. Oza S. Hogan D. Chu Y. Perin J. Zhu J.. (2016). Global, regional, and national causes of under-5 mortality in 2000–15: an updated systematic analysis with implications for the sustainable development goals. Lancet 388, 3027–3035. 10.1016/S0140-6736(16)31593-8 27839855 PMC5161777

[B32] Lubell Y. Blacksell S. D. Dunachie S. Tanganuchitcharnchai A. Althaus T. Watthanaworawit W.. (2015). Performance of C-reactive protein and procalcitonin to distinguish viral from bacterial and malarial causes of fever in Southeast Asia. BMC Infect. Dis. 15:511. 10.1186/s12879-015-1272-6 26558692 PMC4642613

[B33] Manoharan A. Manchanda V. Balasubramanian S. Lalwani S. Modak M. Bai S.. (2017). Invasive pneumococcal disease in children aged younger than 5 years in India: a surveillance study. Lancet Infect. Dis. 17, 305–312. 10.1016/S1473-3099(16)30466-2 27956163

[B34] Ministry of Health and Population (MOHP) New ERA, ICF International (2012). Nepal Demographic and Health Survey 2011. Kathmandu: Ministry of Health and Population, New ERA, and ICF International.

[B35] Mitchell R. Kelly D. F. Pollard A. J. Trück J. (2014). Polysaccharide-specific B cell responses to vaccination in humans. Hum. Vaccin. Immunother. 10, 1661–1668. 10.4161/hv.28350 24632599 PMC5396230

[B36] O'Brien K. L. Baggett H. C. Brooks W. A. Feikin D. R. Hammitt L. L. Higdon M. M. (2019). Causes of severe pneumonia requiring hospital admission in children without HIV infection from Africa and Asia: the PERCH multi-country case-control study. Lancet 394, 757–779. 10.1016/S0140-6736(19)30721-4 31257127 PMC6727070

[B37] O'Brien K. L. Wolfson L. J. Watt J. P. Henkle E. Deloria-Knoll M. McCall N.. (2009). Burden of disease caused by *Streptococcus pneumoniae* in children younger than 5 years: global estimates. Lancet 374, 893–902. 10.1016/S0140-6736(09)61204-6 19748398

[B38] Palkola N. V. Blomgren K. Pakkanen S. H. Puohiniemi R. Kantele J. M. Kantele A. (2016). Immune defense in upper airways: a single-cell study of pathogen-specific plasmablasts and their migratory potentials in acute sinusitis and tonsillitis. PLoS ONE 11:e0154594. 10.1371/journal.pone.0154594 27128095 PMC4851416

[B39] Palkola N. V. Pakkanen S. H. Kantele J. M. Rossi N. Puohiniemi R. Kantele A. (2012). Pathogen-specific circulating plasmablasts in patients with pneumonia. PLoS ONE 7:e34334. 10.1371/journal.pone.0034334 22479603 PMC3314017

[B40] Ramos-Sevillano E. Ercoli G. Brown J. S. (2019). Mechanisms of naturally acquired immunity to *Streptococcus pneumoniae*. Front. Immunol. 10:358. 10.3389/fimmu.2019.00358 30881363 PMC6405633

[B41] Saha S. K. Hossain B. Islam M. Hasanuzzaman M. Saha S. Hasan M.. (2015). Epidemiology of invasive pneumococcal disease in Bangladeshi children before introduction of pneumococcal conjugate vaccine. Pediatr. Infect. Dis. J. 35, 655–661. 10.1097/INF.0000000000001037 26658530

[B42] Sariko M. Anderson C. Mujaga B. S. Gratz J. Mpagama S. G. Heysell S.. (2017). Evaluation of the antibody in lymphocyte supernatant assay to detect active tuberculosis. PLoS ONE 12:e0169118. 10.1371/journal.pone.0169118 28085899 PMC5234774

[B43] Scott J. A. Hall A. J. Dagan R. Dixon J. M. Eykyn S. J. Fenoll A.. (1996). Serogroup-specific epidemiology of *Streptococcus pneumoniae*: associations with age, sex, and geography in 7000 episodes of invasive disease. Clin. Infect. Dis. 22, 973–981. 10.1093/clinids/22.6.973 8783696

[B44] Sheikh A. Bhuiyan M. S. Khanam F. Chowdhury F. Saha A. Ahmed D.. (2009). Salmonella enterica serovar Typhi-specific immunoglobulin A antibody responses in plasma and antibody in lymphocyte supernatant specimens in Bangladeshi patients with suspected typhoid fever. Clin. Vaccine Immunol. 16, 1587–1594. 10.1128/CVI.00311-09 19741090 PMC2772369

[B45] Tang Y. W. Gonsalves S. Sun J. Y. Stiles J. Gilhuley K. A. Mikhlina A. (2016). Clinical evaluation of the Luminex NxTAG respiratory pathogen panel. J. Clin. Microbiol. 54, 1912–1914. 10.1128/JCM.00482-16 27122378 PMC4922127

[B46] Tuerlinckx D. Smet J. De Schutter I. Jamart J. Vergison A. Raes M.. (2013). Evaluation of a WHO-validated serotype-specific serological assay for the diagnosis of pneumococcal etiology in children with community-acquired pneumonia. Pediatr. Infect. Dis. J. 32, e277–e84. 10.1097/INF.0b013e31828c363f 23407099

[B47] Wahl B. O'Brien K. L. Greenbaum A. Majumder A. Liu L. Chu Y.. (2018). Burden of *Streptococcus pneumoniae* and *Haemophilus influenzae* type b disease in children in the era of conjugate vaccines: global, regional, and national estimates for 2000–15. Lancet Glob. Health 6, e744–e757. 10.1016/S2214-109X(18)30247-X 29903376 PMC6005122

[B48] Wu Z. Deloria-Knoll M. Hammitt L. L. Zeger S. L. (2016). Partially latent class models for case-control studies of childhood pneumonia aetiology. Appl. Stat. 65, 97–114. 10.1111/rssc.12101 PMC7169268 32327815

[B49] Zhang Q. Bernatoniene J. Bagrade L. Pollard A. J. Mitchell T. J. Paton J. C.. (2006). Serum and mucosal antibody responses to pneumococcal protein antigens in children: relationships with carriage status. Eur. J. Immunol. 36, 46–57. 10.1002/eji.200535101 16342325

[B50] Zhang Q. Choo S. Finn A. (2002). Immune responses to novel pneumococcal proteins pneumolysin, PspA, PsaA, and CbpA in adenoidal B cells from children. Infect. Immun. 70, 5363–5369. 10.1128/IAI.70.10.5363-5369.2002 12228260 PMC128328

[B51] Zou H. Hastie T. (2005). Regularization and variable selection via the elastic net. J. R. Stat. Soc. B 67, 301–320. 10.1111/j.1467-9868.2005.00503.x

