# Supplementary figures and images for: Assessment of an Antibody-in-Lymphocyte Supernatant Assay for the Etiological Diagnosis of Pneumococcal Pneumonia in Children
Source: Front Cell Infect Microbiol. 2020 Jan 17;9:459. doi: 10.3389/fcimb.2019.00459 (PMC6988833; doi:10.3389/fcimb.2019.00459)

Odds ratio for carriage in pneumonia versus community controls

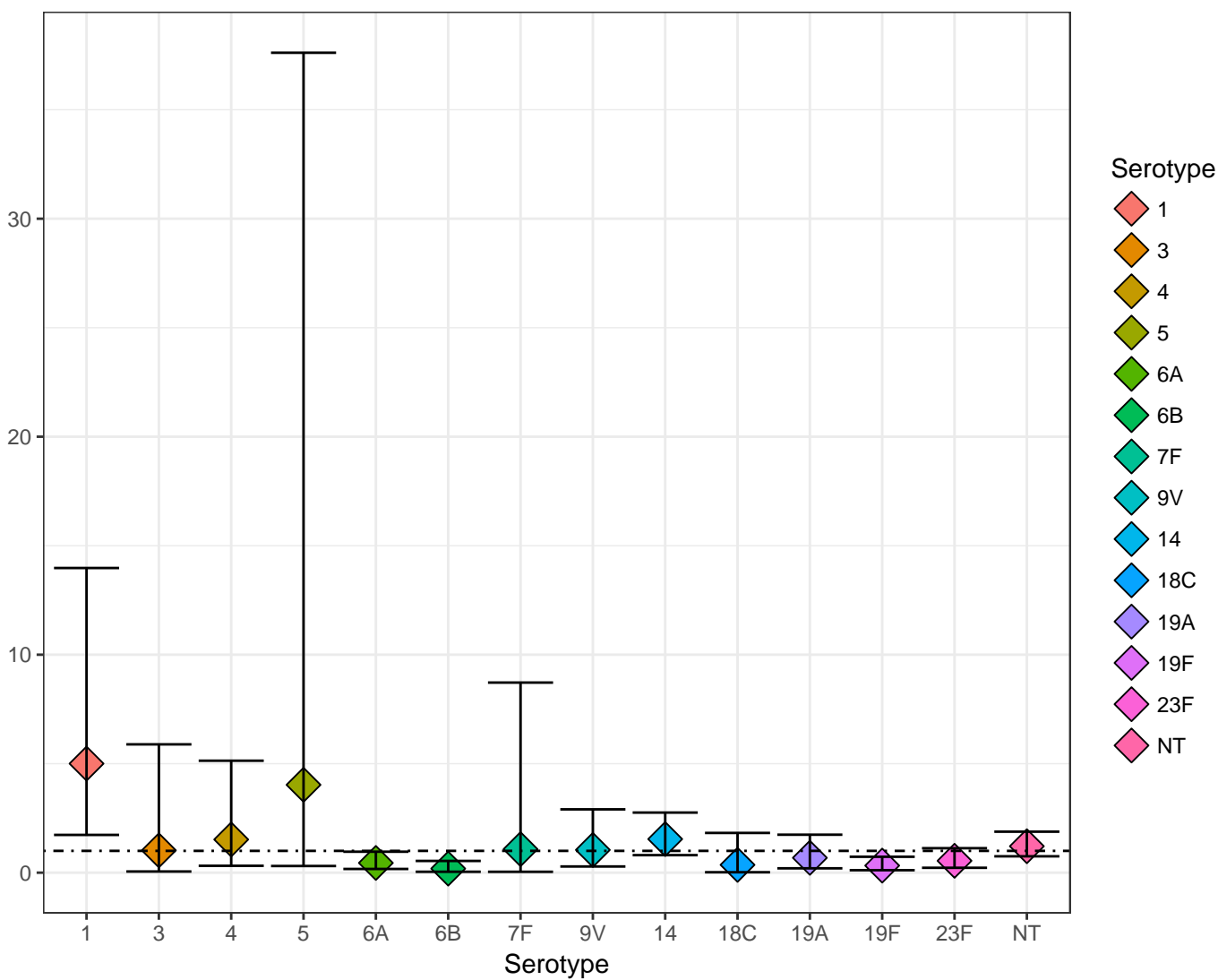

Supplement: Figure S1 — Odds ratios for nasopharyngeal carriage of pneumococcal serotypes contained within 13-valent PCV and non-typeable (NT) pneumococci in pneumonia and community control children, adjusted for age and sex, prior to introduction of the vaccine into the Kathmandu valley. Error bars show 95% confidence intervals. [file Image_1.pdf]

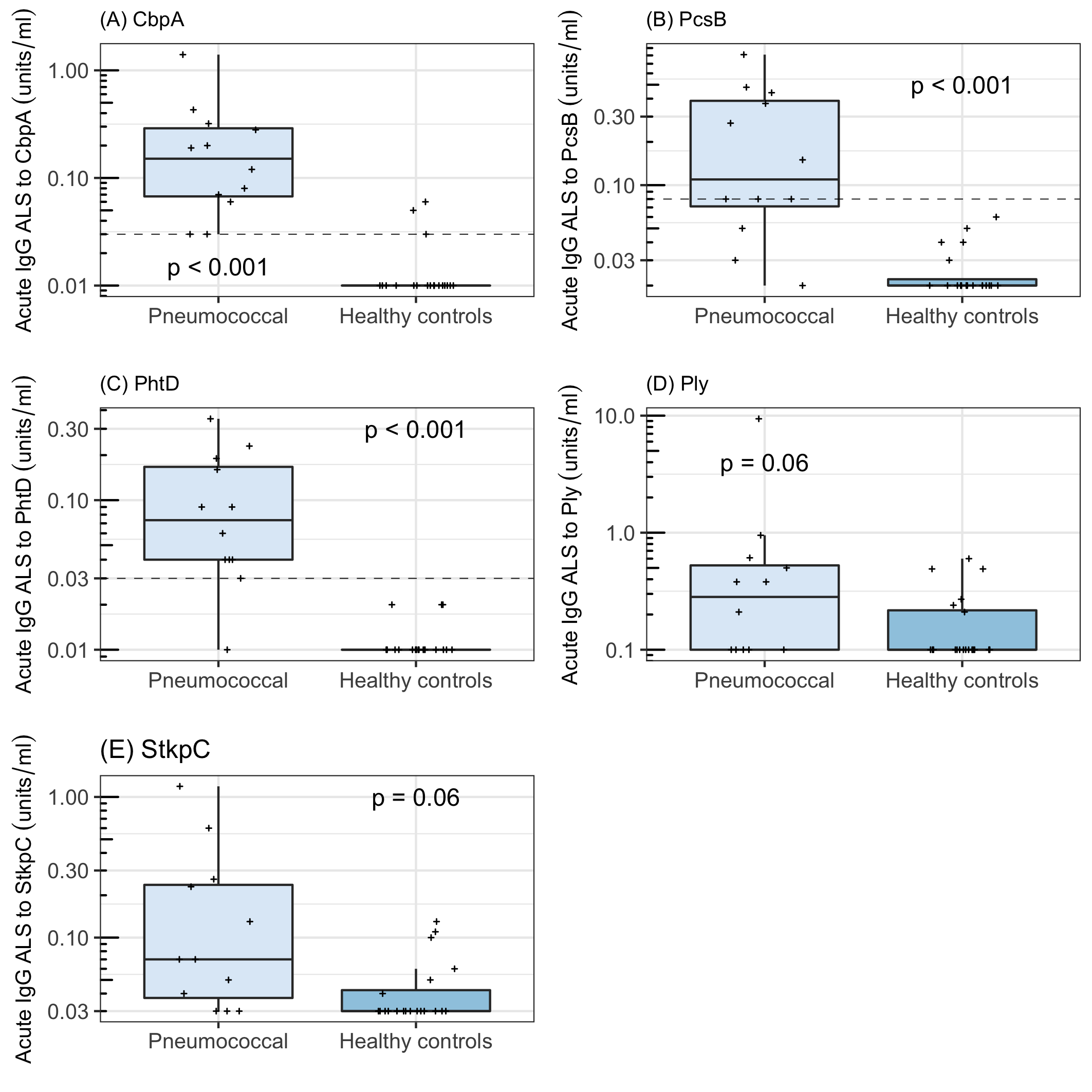

Supplement: Figure S2 — Acute IgG ALS to pneumococcal proteins by children with pneumococcal pneumonia and healthy infant controls. Dashed horizontal lines represent represent thresholds derived from the Youden Index, p-values were derived from the Wilcoxon rank sum test. For all box and whisker plots: the solid line represents the median value, lower hinge 25th centile, upper hinge 75th centile, and whiskers represent 1.5 times the interquartile range. All data points have also been plotted. [file Image_2.tiff]

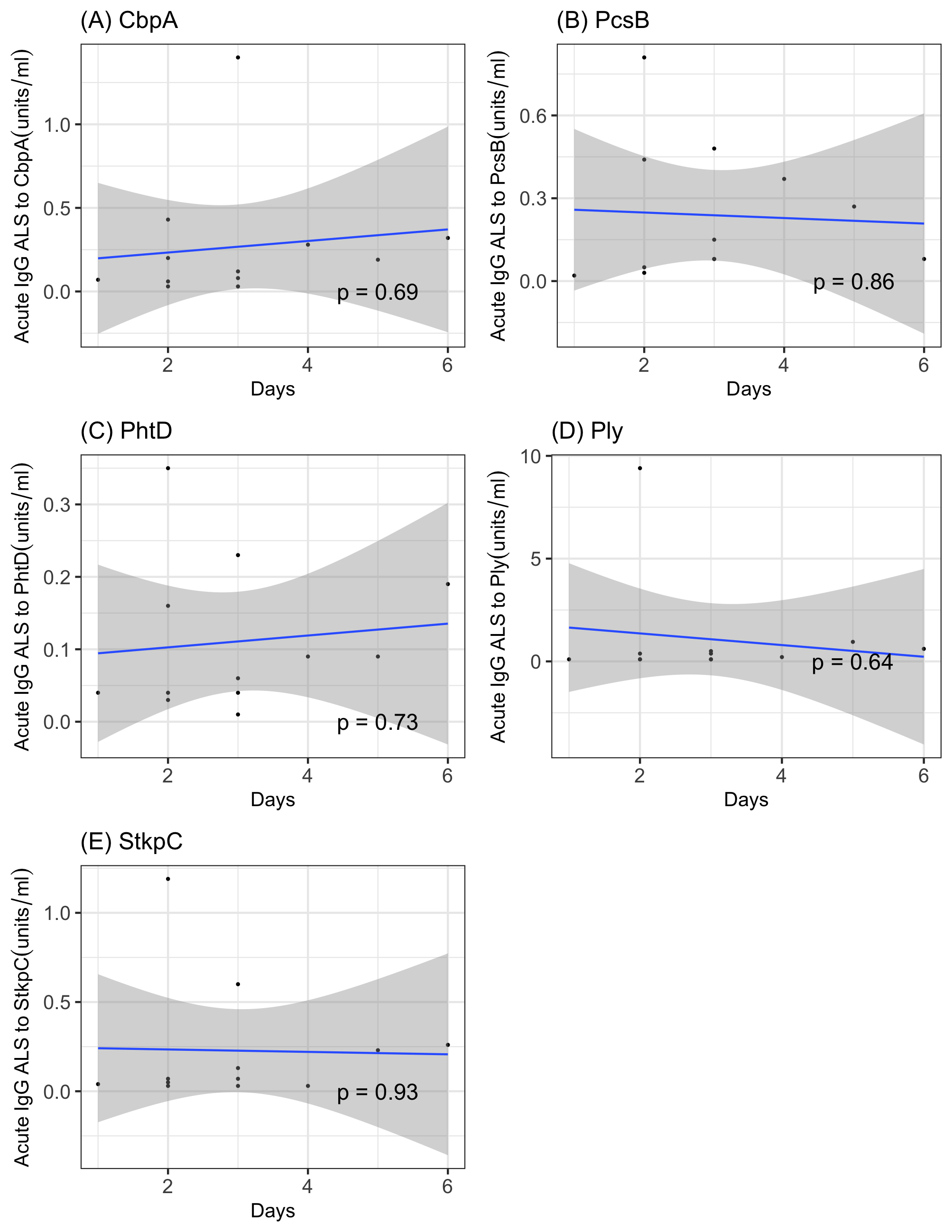

Supplement: Figure S3 — Linear regression analysis of length of illness with acute IgG ALS to pneumococcal proteins (grey colouring represents 95% confidence intervals about the regression line; p-value > 0.5 for all proteins). [file Image_3.tiff]

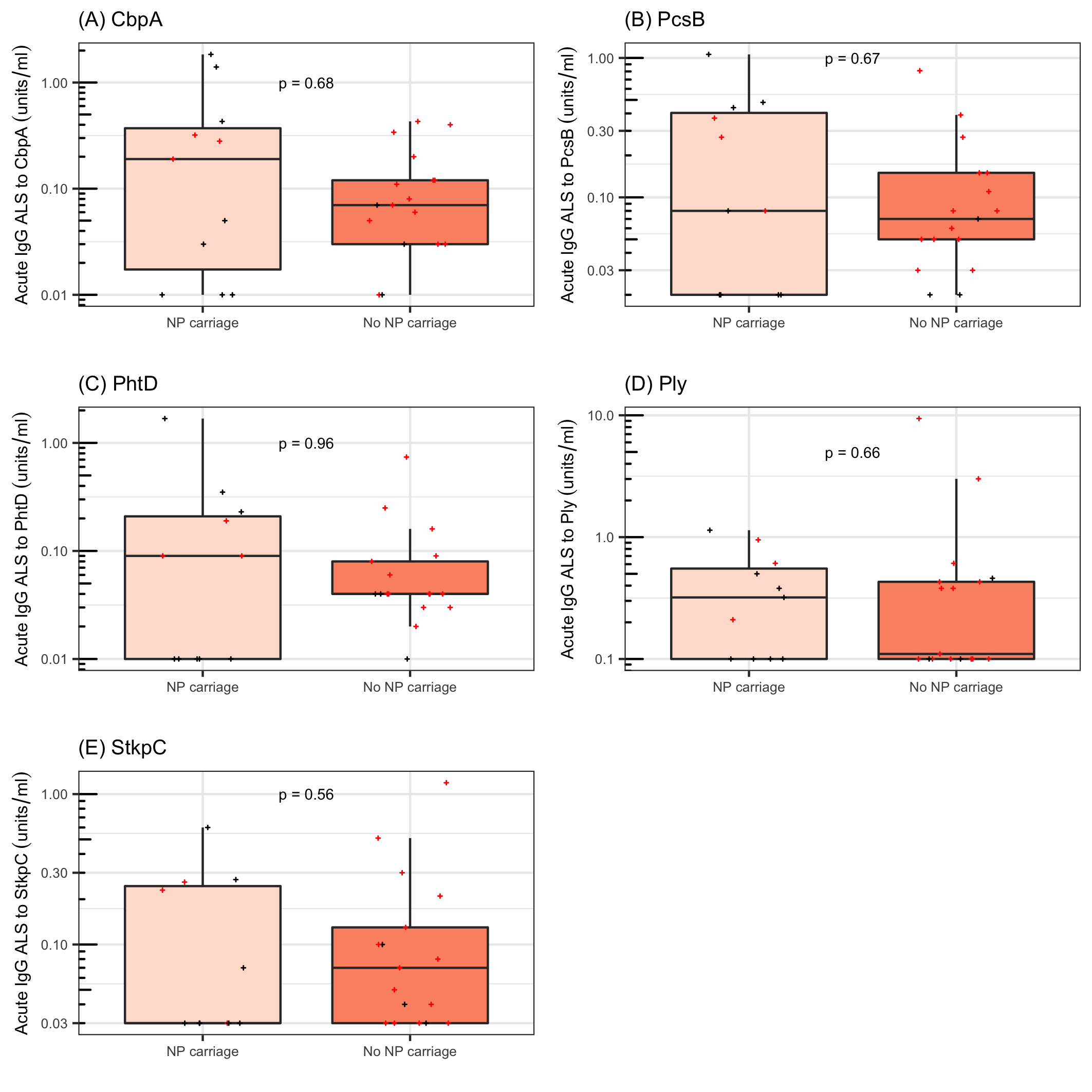

Supplement: Figure S4 — Acute IgG ALS to pneumococcal proteins in children with non-pneumococcal pneumonia by nasopharyngeal carriage of pneumococci. [file Image_4.tiff]
