# Supplementary material for: Assessment of an Antibody-in-Lymphocyte Supernatant Assay for the Etiological Diagnosis of Pneumococcal Pneumonia in Children
Source: Front Cell Infect Microbiol. 2020 Jan 17;9:459. doi: 10.3389/fcimb.2019.00459 (PMC6988833; doi:10.3389/fcimb.2019.00459)
Supplement: Supplementary file 5 [file Table_1.docx]

| **Characteristic** | **IPD** | **No IPD** | **p** | **EPC** | **No EPC** | **p** | **CRP ≥60 mg/l** | **CRP <60 mg/l** | **p** |
| --- | --- | --- | --- | --- | --- | --- | --- | --- | --- |
| n | 8 | 340 |  | 122 | 226 |  | 78 | 270 |  |
| Age (years; median, IQR) | 5.6 (4.2–8.1) | 1.3 (0.6–2.6) | <0.001^1^ | 2.4 (0.9–5.1) | 1.1 (0.6–2.1) | <0.001^1^ | 4.4 (1.9–6.8) | 1.1 (0.6–2.1) | <0.001^1^ |
| 2–11 months | 0 | 136 (40%) |  | 34 (28%) | 102 (46%) |  | 10 (13%) | 126 (47%) |  |
| 1–<2 years | 0 | 82 (24%) |  | 20 (17%) | 62 (28%) |  | 11 (14%) | 71 (26%) |  |
| ≥2–<5 years | 3 (38%) | 83 (25%) |  | 35 (29%) | 51 (23%) |  | 31 (40%) | 55 (21%) |  |
| ≥5–14 years | 5 (62%) | 36 (11%) |  | 32 (26%) | 9 (4.0%) |  | 25 (32%) | 16 (6.0%) |  |
| Female sex | 1 (13%) | 139 (41%) | 0.15^2^ | 55 (45%) | 85 (38%) | 0.21^6^ | 29 (37%) | 111 (41%) | 0.62^6^ |
| Length of illness (days; median, IQR) | 2.5 (2–3) | 4 (3–6) | 0.03^1^ | 5 (3–7) | 4 (3–5) | 0.01^1^ | 4 (2.3–6) | 4 (3–6) | 0.61^1^ |
| Prior antibiotic use | 6 (75%) | 156 (46%) | 0.27^2^ | 64 (52%) | 98 (43%) | 0.16^6^ | 38 (49%) | 124 (46%) | 0.87 |
| NP pneumococcal carriage^7^ | 2 (25%) | 101 (30%) | 1^2^ | 39 (32%) | 64 (28%) | 0.56^6^ | 30 (38%) | 73 (27%) | 0.07^6^ |
| NP pneumococcal carriage (serotype 1) | 1 (13%) | 5 (1.5%) | 0.13^2^ | 4 (3.3%) | 2 (0.9%) | 0.23^2^ | 5 (6.4%) | 1 (0.3%) | 0.003^2^ |
| Invasive pneumococcal disease | 8 | 0 | – | 8 (6.5%) | 0 | <0.001^2^ | 5 (6.4%) | 3 (1.1%) | 0.02^2^ |
| Other invasive disease | 0 | 6 (1.7%)^3^ | – | 3 (2.5%) | 3 (1.3%) | 0.42^2^ | 3 (3.8%) | 3 (1.1%) | 0.13^2^ |
| Endpoint consolidation | 8 (100%) | 114 (34%)^4^ | <0.001^2^ | 122 | 0 | – | 58 (74%) | 64 (24%) | <0.001^6^ |
| CRP (mg/l; median, IQR) | 141 (56–183) | 16 (4.0–51) | <0.001^5^ | 56 (15–152) | 10 (2.7–25.3) | <0.001^5^ | 118 (86, 184) | 10 (2.7–22) |  |
| CRP ≥60 mg/l | 3 (38%) | 73 (21%) |  | 58 (48%) | 20 (8.8%) |  | 78 | 0 |  |
| NP RSV carriage^8^ |  |  |  |  |  |  | 3 (4.1%) | 28 (11%) | 0.07^6^ |
| NP other viral carriage^8^ |  |  |  |  |  |  | 4 (5.5%) | 23 (9.4%) | 0.07^6^ |

**Table S1.** Clinical characteristics of children enrolled into the study by the key endpoints: invasive pneumococcal disease (IPD) status, radiographic endpoint consolidation, and plasma CRP concentration ≥60 mg/l.
