# Supplementary material for: Assessment of an Antibody-in-Lymphocyte Supernatant Assay for the Etiological Diagnosis of Pneumococcal Pneumonia in Children
Source: Front Cell Infect Microbiol. 2020 Jan 17;9:459. doi: 10.3389/fcimb.2019.00459 (PMC6988833; doi:10.3389/fcimb.2019.00459)
Supplement: Supplementary file 6 [file Table_2.docx]

**Table S2.** Diagnostic accuracy for acute IgG ALS to pneumococcal proteins to discriminate between *pneumococcal pneumonia* and healthy control infants in Nepal.

| *Pneumococcal pneumonia* versus healthy controls | | | | | |
| --- | --- | --- | --- | --- | --- |
|  | **CbpA** | **PcsB** | **PhtD** | **Ply** | **StkpC** |
| Cut-off value | 0.03 | 0.08 | 0.03 | 0.38 | 0.04 |
| Sensitivity | 1.0 (0.74–1.0) | 0.75 (0.43–0.95) | 0.92 (0.62–1.0) | 0.50 (0.21–0.79) | 0.75 (0.43–0.95) |
| Specificity | 0.85 (0.62–0.97) | 1.0 (0.83–1.0) | 1.0 (0.83–1.0) | 0.85 (0.62–0.97) | 0.70 (0.46–0.88) |
| AUROCC | 0.98 (0.94–1.0) | 0.92 (0.81–1.0) | 0.95 (0.86–1.0) | 0.68 (0.49–0.87) | 0.77 (0.60–0.94) |
| AUROCC, area under the receiver-operating characteristic curve. Cutpoint value expressed as units/ml. | | | | | |
